# Cuticular competing endogenous RNAs regulate insecticide penetration and resistance in a major agricultural pest

**DOI:** 10.1186/s12915-023-01694-z

**Published:** 2023-09-05

**Authors:** Li-Wei Meng, Guo-Rui Yuan, Meng-Ling Chen, Li-Sha Zheng, Wei Dou, Yu Peng, Wen-Jie Bai, Zhen-Yu Li, John Vontas, Jin-Jun Wang

**Affiliations:** 1 Key Laboratory of Entomology and Pest Control Engineering, College of Plant Protection, Southwest University, Chongqing, 400716 China; 2 Key Laboratory of Agricultural Biosafety and Green Production of Upper Yangtze River (Ministry of Education), Academy of Agricultural Sciences, Southwest University, Chongqing, 400715 China; 3 Institute of Molecular Biology and Biotechnology, Foundation for Research and Technology-Hellas, 70013 Heraklion, Greece; 4 Pesticide Science Laboratory, Department of Crop Science, Agricultural University of Athens, Athens, 11855 Greece

**Keywords:** microRNA, Cuticular protein, Long noncoding RNA, Insecticide resistance, Insect integument

## Abstract

**Background:**

The continuously developing pesticide resistance is a great threat to agriculture and human health. Understanding the mechanisms of insecticide resistance is a key step in dealing with the phenomenon. Insect cuticle is recently documented to delay xenobiotic penetration which breaks the previous stereotype that cuticle is useless in insecticide resistance, while the underlying mechanism remains scarce.

**Results:**

Here, we find the integument contributes over 40.0% to insecticide resistance via different insecticide delivery strategies in oriental fruit fly. A negative relationship exists between cuticle thickening and insecticide penetration in resistant/susceptible, also in field strains of oriental fruit fly which is a reason for integument-mediated resistance. Our investigations uncover a regulator of insecticide penetration that miR-994 mimic treatment causes cuticle thinning and increases susceptibility to malathion, whereas miR-994 inhibitor results in opposite phenotypes. The target of miR-994 is a most abundant cuticle protein (CPCFC) in resistant/susceptible integument expression profile, which possesses capability of chitin-binding and influences the cuticle thickness-mediated insecticide penetration. Our analyses find an upstream transcriptional regulatory signal of miR-994 cascade, long noncoding RNA (*lnc19419*), that indirectly upregulates *CPCFC* in cuticle of the resistant strain by sponging miR-994. Thus, we elucidate the mechanism of cuticular competing endogenous RNAs for regulating insecticide penetration and demonstrate it also exists in field strain of oriental fruit fly.

**Conclusions:**

We unveil a regulatory axis of *lnc19419* ~ miR-994 ~ *CPCFC* on the cuticle thickness that leads to insecticide penetration resistance. These findings indicate that competing endogenous RNAs regulate insecticide resistance by modulating the cuticle thickness and provide insight into the resistance mechanism in insects.

**Supplementary Information:**

The online version contains supplementary material available at 10.1186/s12915-023-01694-z.

## Background

The oriental fruit fly *Bactrocera dorsalis* (Hendel) is one of the most destructive pest of fruits and vegetables around the world [1], which could cost as much as $3 million from annual losses in major fruit crops in Hawaii and the number is estimated $44–176 million in California, US [2]. Chemical insecticides are primarily used to control this pest in the field. However, insects rapidly evolve resistance to insecticides, which is a challenge for sustainable pest management. Various adaptive mechanisms have been described, such as enhanced metabolism, target-site insensitivity, and reduced penetration, while the penetration resistance is usually embodied in primary stage of xenobiotics such as insecticide entry and is mediated by the insect cuticle as the first defensive barrier [3]. The main components of the cuticle are chitin and cuticular proteins (CPs) [4–7]. However, it remains unclear how these various components interact to increase insecticide resistance, and the regulatory mechanisms are remained unknown [8].

MicroRNAs (miRNAs) are regulators of gene expression at the post-transcriptional level that are known to participate in multiple physiological processes in insects, including development, reproduction, and adaptation to environmental stressors such as insecticides [9–11]. Most miRNAs are negative regulators and the general mechanism involves direct interaction with target messenger RNAs (mRNAs) and long non-coding RNAs (lncRNAs) [9, 12]. Insect lncRNAs are reported to be associated with insecticide resistance, behavioral plasticity, and reproduction [13–16], however, the regulatory mechanism of lncRNAs in insects still remains scarce [17–19]. For example, the cooperative action of lncRNAs and miRNAs in transcriptional regulations was investigated in *Plutella xylostella*, and their expression has been consequently proposed as a strategy for the management of agricultural pests [19].

The oriental fruit fly, *B. dorsalis* is renowned for its resistance to multiple insecticides, which prevents effective and sustainable pest management [20, 21]. Initial studies have focused on *B. dorsalis* detoxification enzymes [22, 23] and insecticide targets [24], but this was not sufficient to explain the strong resistance phenotype indicating that additional mechanisms such as penetration resistance might also be involved [25, 26]. Here, we used insecticide penetration assays, different insecticide delivery strategies, transmission electron microscopy (TEM) and silencing of the chitin synthetase gene (*CHS1*) to confirm that *B. dorsalis* gains penetration resistance to the insecticide malathion through the thickening of the cuticle. We then identified miR-994 as a mediator of penetration resistance by small RNA sequencing (RNA-Seq), RT-qPCR analysis, mimic/inhibitor injection, TEM, insecticide penetration assays and bioassays. We used bioinformatics analysis, RNA-Seq, RNA pull-down assays, dual luciferase assays and fluorescence signal colocalization to confirm that miR-994 is negatively regulated by *lnc19419* and in turn negative regulates the cuticular protein CFC (*CPCFC*). We confirmed ability of *lnc19419* and *CPCFC* to promote cuticular thickening and malathion resistance by RNA interference (RNAi), and also established the regulatory interactions between *lnc19419*, miR-994 and *CPCFC* mRNA. These findings provide insight into the role of non-coding RNAs and *CPCFC* in cuticle-mediated insecticide resistance and contribute to elucidate underlying mechanisms during the long-term insecticide exposures.

## Results

### Penetration resistance in *B. dorsalis* is mediated by cuticular thickening

**Fig. 1:**
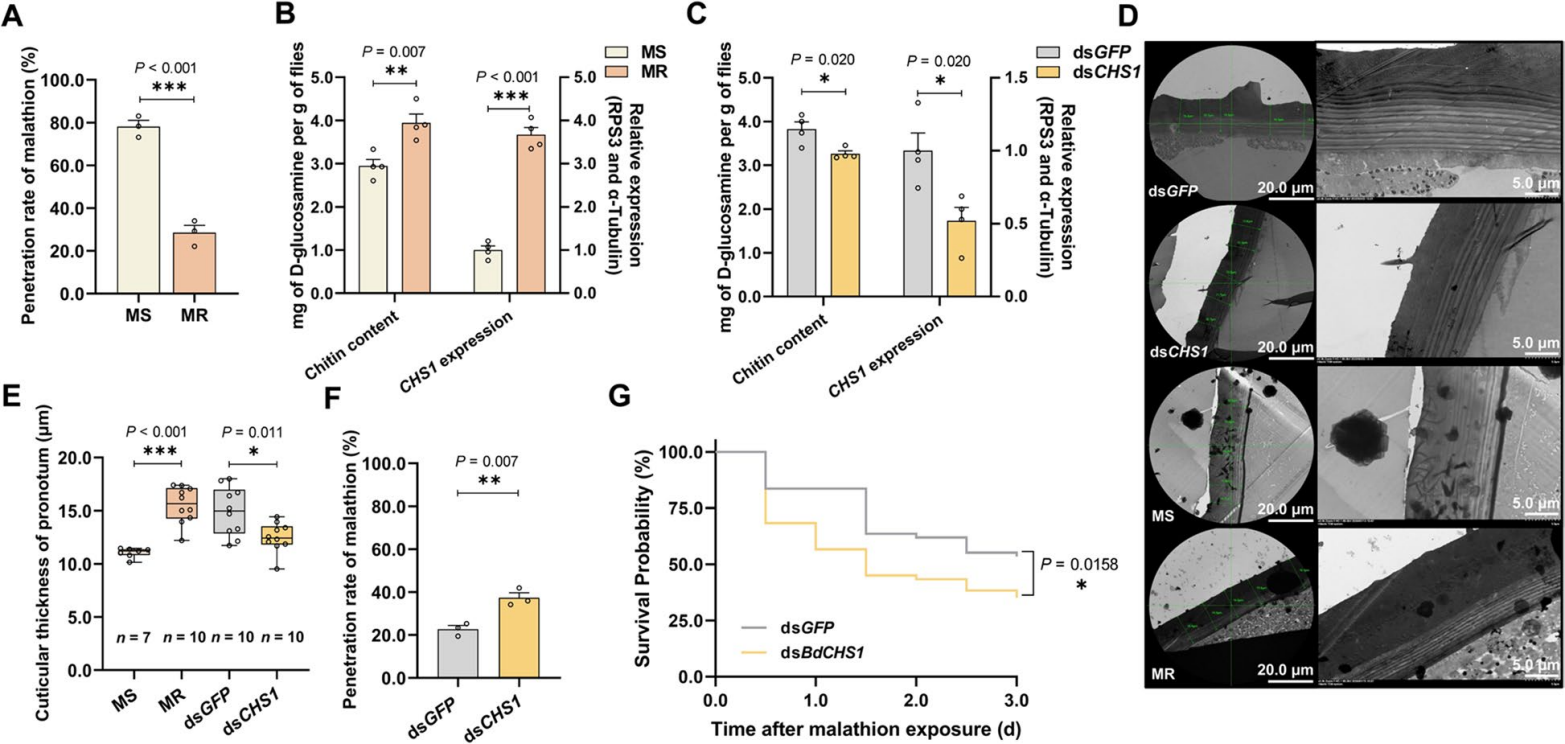
Cuticular thickening mediates malathion penetration resistance in *B. dorsalis*. **A** Penetration rate of malathion in MR and MS strains. Each eluant of MR and MS strains contained 30 and 50 5-day-old adult flies, respectively (*n* = 3). **B** Chitin content and expression of *CHS1* in MR and MS strains. Each sample contained six 5-day-old adult flies (*n* = 4). **C** The chitin content (mg of D-glucosamine per g of flies) and expression of *CHS1* was analyzed after silencing *CHS1*. Each sample contained six 5-day-old adult flies. Data are means ± SEM (*n* = 4). **D** Ultrastructure of the pronotum cuticle in MR, MS, ds*GFP* and ds*CHS1* groups. **E** Pronotum cuticular thickness in strains MR (*n* = 10), MS (*n* = 7) and after silencing *CHS1* (*n* = 10). Each thickness value is the mean of five randomly selected cross-sectional measurements. **F** Penetration rate of malathion after silencing *CHS1*. Each sample contained 30 5-day-old adult flies (*n* = 3). **G** The effect of *CHS1* silencing on malathion susceptibility in *B. dorsalis*. Survival rate was observed at 0–3 day after malathion exposure (*n* = 120)

The topical application of malathion repetitively on the pronotum of *B. dorsalis* was used to select the resistance over 26 generations, resulting in the emergence of a malathion-resistant (MR) strain with an LD_50_ of up to 3452.5 ng/fly. This was 53.64-fold higher than the LD_50_ of the malathion susceptible (MS) strain (64.37 ng/fly), which had never been exposed to the insecticide (Table 1). To determine whether resistance was conferred by the cuticle, we compared the toxicity of topical and injected malathion (the latter bypassing the cuticle) in MR and MS flies (Additional file 1: Fig. S1A). The LD_50_ of MS adults decreased from 64.37 to 44.11 ng/fly after direct injection whereas that of the MR adults decreased from 3452.50 to 1333.77 ng/fly (Table 1). The injection of malathion increased the susceptibility of both strains, but the topical/injected ratios were 1.46-fold for the MS strain and 2.58-fold for the MR strain, confirming that the cuticle played a critical role in the evolution of malathion resistance during continuous selection (Table 1). The penetration ratio in the MR strain at 1 h post-exposure was 28.5% compared to 78.2% for the MS strain (Fig. 1A). The cuticle of the MR strain was therefore able to block the insecticide more effectively.

**Table 1:** Toxicity of malathion against the MR and MS strains of *B. dorsalis*

Strains	LD_50_ (95% ng/fly)	Y = LD – P line	χ^2^	Resistance ratio (RR)	Topical/injected ratio
**Topical application**					
MS	64.37 (56.18–73.40)	Y = 3.04X – 6.41	3.63	-	-
MR	3452.50 (3081.60–3890.15)	Y = 3.11X – 11.95	1.07	53.64^a^	-
**Injection**					
MS	44.11 (38.15–58.97)	Y = 5.92X – 9.74	6.77	0.69^b^	1.46^d^
MR	1,333.77 (1281.53–1383.49)	Y = 8.57X – 26.78	3.03	30.24^c^	2.59^e^

LD_50_, dosage resulting in the death of 50% of the test animals. Topical application means that malathion was applied to the pronotum using a PB600-1 repeating dispenser. Injection means that the malathion was injected into the body of the fly through the thorax

^a^LD_50_ of MR topical application/MS topical application

^b^LD_50_ of MS injection/MS topical application

^c^LD_50_ of MR injection/MS injection

^d^LD_50_ of MS topical application/MS injection

^e^LD_50_ of MR topical application/MR injection

The chitin content of the MR strain was significantly higher than that of the MS strain with 1.34-fold (Fig. 1B). The pronotum cuticle (the site of insecticide exposure) was also significantly thicker with 1.41-fold in the MR strain (Figs. 1D and E). Cuticular thickness therefore correlates with cuticle-mediated insecticide resistance in *B. dorsalis*. To investigate the mechanisms of cuticular thickness in malathion resistance, we analyzed the expression of the chitin synthetases *CHS1a*, *CHS1b* and *CHS2*. The *CHS1a* and *CHS1b* sequences were highly similar, and were also expressed in a similar pattern which was upregulated in MR strains (Additional file 1: Figs. S1B and S1C). We therefore designed qPCR primers and dsRNA constructs spanning the common region of these homologous genes to investigate the function of *CHS1* (Fig. 1B). The knockdown of *CHS1* by RNAi resulted in a lower chitin content and a thinner cuticle with decrease of approximate 14.6% and 16.2%, respectively, compared to the control (Figs. 1C and E). The thinner pronotum cuticle caused increased penetration of malathion with 14.7% (Fig. 1F) and enhanced mortality with 25.0% post malathion treatment 72 h (Fig. 1G). These data demonstrated that pronotum cuticular thickening contributed to the selected malathion resistance phenotype in *B. dorsalis*.

### miR-994 is a potential regulator of malathion resistance

To investigate the potential role of small RNAs in the regulation of penetration resistance by cuticular thickening in *B. dorsalis*, we sequenced the small RNA transcriptome of the MR and MS strains. We recovered 76 known and 221 novel miRNAs (Additional File 2: Data S1), 10 of which were downregulated in the MR strain (Additional file 1: Figs. S2A and S2B, and Additional File 2: Data S2). The expression profiles of the 10 miRNAs were validated by qPCR and in most cases agreed with the RNA-Seq data (Additional file 1: Fig. S2C). However, six of them showed high log_2_ FC values but less than 1,000 transcripts per million (TPM), indicating low expression levels. The remaining four (miR-6, miR-286, miR-994 and miR-318) were expressed at higher levels (more than 1,000 TPM) (Additional File 1: Fig. S2B), but miR-994 was the only miRNA with pronounced up-regulation in the pronotum cuticle of the MS strain compared to the MR strain (Additional File 1: Figs. S2C and S2D). These data suggested that miR-994 was the most promising candidate in regulating resistance.

### miR-994 regulates the thickness of the pronotum cuticle

**Fig. 2:**
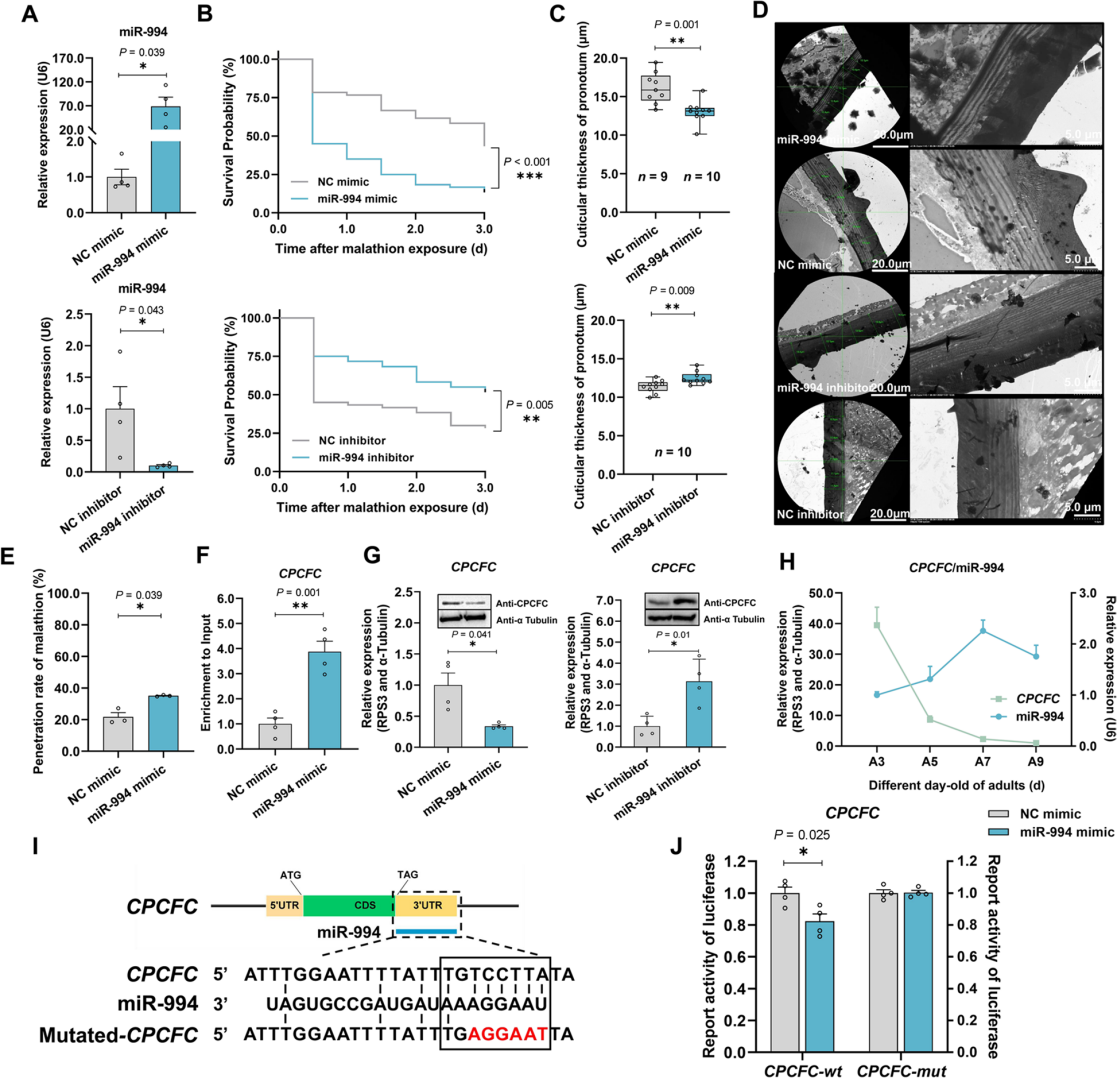
Malathion penetration resistance is mediated by miR-994, which modulates cuticular thickness via *CPCFC*. **A** Expression analysis of miR-994 after injecting synthetic miR-994 or an inhibitor (*n* = 4). **B** *B. dorsalis* susceptibility to malathion after injecting synthetic miR-994 or an inhibitor into MR and MS strains, respectively. Survival rate was observed at 0–3 day after malathion exposure (*n* = 120). **C** Pronotum cuticular thickness after injecting synthetic miR-994 (*n* = 10), negative control (NC) miRNA (*n* = 9) or corresponding inhibitors (*n* = 10). The synthetic miR-994 and NC were injected into MR flies and the inhibitor into MS flies. Each thickness value is the mean of five randomly selected cross-sectional measurements. **D** Ultrastructure of the pronotum cuticle following the injection of synthetic miR-994 or an inhibitor. **E** Malathion penetration rate after injecting synthetic miR-994. Each eluant contained 30 5-day-old adult flies (*n* = 3). **F** Enrichment analysis (biotin–avidin RNA pull-down) following the binding of miR-994 to *CPCFC* (*n* = 4). (G) Expression of *CPCFC* after injecting synthetic miR-994 or an inhibitor (*n* = 4). **H** Expression profiles of miR-994 and *CPCFC* in adult flies at 3, 5, 7 and 9 days old (A3, A5, A7 and A9, *n* = 4). **I** Target site analysis, showing the interaction between miR-994 and *CPCFC* mRNA. Mutated *CPCFC* contained the sequence 5′-AGGAAT-3′ (red). **J** Luciferase activity indicating the targeting of *CPCFC* by miR-994 in vitro. *CPCFC*-*wt* and *CPCFC*-*mut* indicate HEK293-T cells containing the 3′ UTR of wild-type and mutated *CPCFC*, respectively (*n* =4)

To confirm our hypothesis, we injected the MR strain with synthetic miR-994 and the MS strain with a miR-994 inhibitor. In the MR strain, miR-994 expression increased by 65.0-fold, enhancing its susceptibility to malathion significantly by 30.0% after 72 h (Figs. 2A and B). In contrast, injecting the miR-994 inhibitor into the MS strain increased malathion resistance (Figs. 2A and B). These data confirmed that miR-994 is associated with susceptibility to malathion in *B. dorsalis*.

We also measured the activity of detoxification enzymes following the injection of synthetic miR-994 and observed no changes in activity (Additional File 3: Table S1). However, the penetration of malathion increased significantly by 13.4% after injection, providing additional evidence that miR-994 regulates penetration resistance (Fig. 2E). After injecting synthetic miR-994 into the MR strain, the pronotum cuticle became significantly thinner with 19.1%, whereas the miR-994 inhibitor caused the pronotum cuticle to become thicker in the MS strain (Figs. 2C and D). These results confirmed our hypothesis that miR-994 expression confers susceptibility to malathion by reducing the thickness of the pronotum cuticle.

### The target of miR-994 is *CPCFC* mRNA

The target of miR-994 was determined by RNA-Seq analysis of the MR strain following the injection of synthetic miR-994. Given the negative regulatory mechanism of most miRNAs, we focused on the 186 genes (Additional File 2: Data S3) that were downregulated by miR-994 treatment (Additional File 1: Fig. S3A). It contained several potential resistant-related genes including detoxification enzymes, CPs and *CHS1*. These genes were compared to the RNA-Seq data from the pronotum of the MR and MS strains to narrow the range of targets. Among the 480 genes (Additional File 2: Data S4) that were upregulated in the MR strain compared to the MS strain (Additional File 1: Fig. S3D), we found 20 that were also downregulated by the injection of synthetic miR-994 (Additional File 1: Fig. S3B and S3C). Using four different target prediction algorithms, this list of 20 was narrowed to four mRNAs encoding CPCFC, CHS1, major royal jelly protein (MRJP), and an uncharacterized protein (Additional File 1: Fig. S3F).

We used a biotin–avidin RNA pull-down system to confirm the target gene of miR-994 among the four candidates. Compared with the negative control, the *CPCFC* mRNA was significantly enriched by 3.9-fold, whereas *CHS1*, *MRJP*, and the mRNA encoding the uncharacterized protein were unchanged, indicating no interaction with miR-994 (Fig. 2F and Additional File 1: Fig. S3G). Fluorescence in situ hybridization showed that the signals of miR-994^Cy3^ and *CPCFC*^Fam^ were co-localized in the pronotum cuticle of MS strain (Additional File 1: Fig. S3H), which was consistent with our qPCR results. The expression profiles of the miR-994 and *CPCFC* genes were complementary in adults of different ages (Fig. 2H). Furthermore, miR-994 overexpression in the MR strain caused the 56.8% depletion of *CPCFC* mRNA and also reduced the abundance of the CPCFC protein (Fig. 2G and Additional File 1: Fig. S4A), whereas the inhibition of miR-994 in the MS strain increased *CPCFC* mRNA and CPCFC protein levels (Fig. 2G and Additional File 1: Fig. S4C). These in vivo assays supported the negative regulatory relationship between miR-994 and *CPCFC*. Finally, a dual-luciferase reporter assay showed that relative luciferase activity decreased significantly by 17.7% following the co-transfection of pmirGLO-*CPCFC* and the synthetic miR-994 (Fig. 2J), compared with pmirGLO-*CPCFC* plus a negative control miRNA. A plasmid with mutated target sites in *CPCFC* (5′-AGGAAT-3′ instead of 5′-TCCTTA-3′) was also co-transfected with the synthetic miR-994 and did not induce a change in luciferase activity (Figs. 2I and J). These in vitro results confirmed that miR-994 negatively regulates the expression of *CPCFC* by binding to the 3′ untranslated region (3′-UTR) at the sequence 5′-TGTCCTTA-3′ (Fig. 2I).

### CPCFC is a critical component in cuticle-mediate penetration resistance

**Fig. 3:**
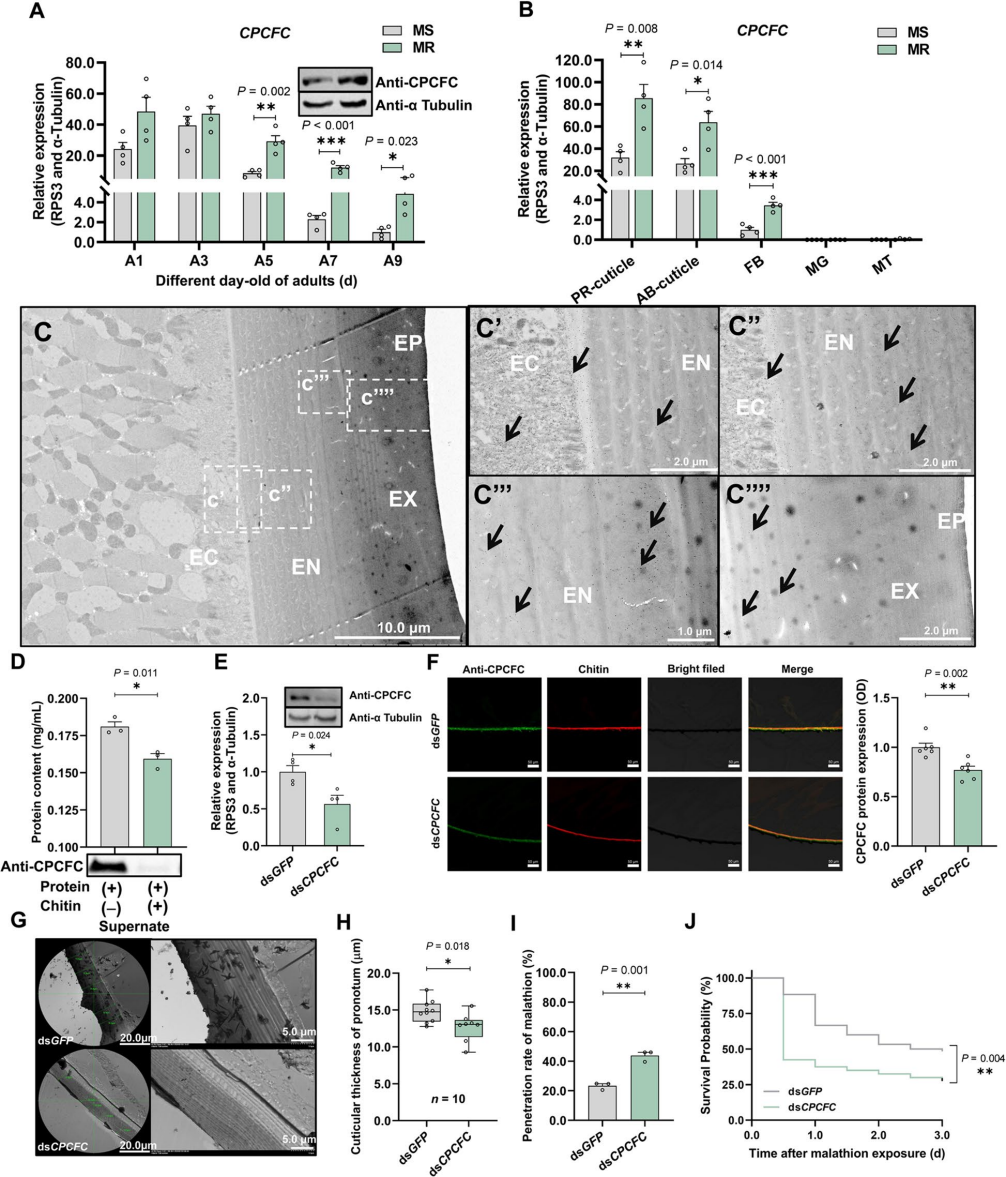
CPCFC is necessary for cuticle-mediated malathion penetration resistance. **A** The expression profile of *CPCFC* in MS and MR adult flies at 3, 5, 7 and 9 days old (A3, A5, A7 and A9, *n* = 4). **B** The expression levels of *CPCFC* in different tissues of the MR and MS strains: pronotum (PR) cuticle, abdomen (AB) cuticle, fat body (FB), midgut (MG) and Malpighian tubule (MT). Each sample contained 20 fly-tissues of 5-day-old adult (*n* = 4). **C** Immunolocalization of CPCFC on ultrathin sections of the pronotum cuticle, showing the epicuticle (EP), exocuticle (EX), endocuticle (EN) and epidermic cells (EC). The white squares correspond to zoomed cuticular ultrastructure of (C′), (C′′), (C′′′) and (C′′′′). The black arrow indicates the 10-nm colloidal gold signal of CPCFC. (C′), (C′′) and (C′′′) The pronotum cuticular ultrastructure of EC and EN. (C′′′′) The pronotum cuticular ultrastructure of EX and EP. **D** Chitin-binding activity of CPCFC, showing the protein concentration in the supernatant (*n* = 3). **E** *CPCFC* expression levels after silencing (*n* = 4). **F** The effects of *CPCFC* silencing on the structure of the pronotum cuticle. CPCFC protein expression was determined by the fluorescence signal intensity over length in each sample (*n* = 6). **G** Analysis of the cuticular ultrastructure after silencing *CPCFC*. **H** Analysis of the pronotum cuticular thickness after silencing *CPCFC* (*n* = 10). Each thickness value is the mean of five randomly selected cross-sectional measurements. **I** The malathion penetration rate after silencing *CPCFC*. Each eluant contained 30 5-day-old adult flies (*n* = 3). **J** The effect of *CPCFC* silencing on susceptibility to malathion. Survival rate was observed at 0–3 day after malathion exposure (*n* = 120)

We assessed the expression of *CPCFC* gene and CPCFC protein in 5-day-old adults of each strain, revealing 3.4-fold higher mRNA levels and also higher protein levels in the MR strain (Fig. 3A and Additional File 1: Fig. S4B). *CPCFC* was upregulated in multiple tissues of the MR strain compared to the MS strain, including a 2.7-fold increase in the pronotum cuticle (Fig. 3B). This was consistent with our RNA-Seq data in the same tissues, supporting the conclusion that *CPCFC* is involved in the malathion resistance phenotype (Additional File 1: Fig. S3E). Immunohistochemistry and immunogold staining followed by laser scanning confocal microscopy (LSCM) and TEM revealed that CPCFC and chitin were enriched in the cuticle (Additional File 1: Fig. S4I) and the colloidal gold signal was also enriched in the cuticle chitin assembly (Fig. 3C) including the endocuticle and epidermic cells (Figs. 3C’ and C’’), but not the exocuticle (Figs. 3C’’’ and C’’’’).

Cuticle proteins are usually able to bind chitin and thus modulate cuticular structure and thickness. We therefore expressed recombinant CPCFC in vitro (Additional File 1: Fig. S4G) to evaluate its chitin-binding ability (Additional File 1: Fig. S4H). Western blot analysis showed that the CPCFC signal was barely detectable in the supernatant after incubation with chitin resin, indicating that CPCFC was bound to chitin and thus pulled into the pellet (Fig. 3D and Additional File 1: Fig. S4H). In addition, the protein concentration in the supernatant was significantly lower (11.9%) compared with the control without chitin resin (Fig. 3D).

To determine the function of *CPCFC* in cuticular development and insecticide resistance, we silenced the *CPCFC* gene by RNAi (43.6% for mRNA; 23.1% for protein in pronotum cuticle) (Figs. 3E, F and Additional File 1: Fig. S4E). The pronotum cuticle became 14.2% thinner after *CPCFC* silencing (Figs. 3G and H), which increased the penetration of malathion by 20.5% (Fig. 3I), resulting in a 30.0% increase in mortality 72 h after treatment (Fig. 3J). These data indicate that *CPCFC* is a component of the pronotum cuticle that modulates its thickness and determines the degree of malathion penetration resistance.

### *lnc19419* sequesters miR-994 to modulate penetration resistance

Because lncRNAs can act as sponges to sequester miRNAs, we sequenced 6,621 cuticular lncRNA transcripts (Additional File 1: Fig. S5A) and classified them as lincRNAs, antisense lncRNAs, lncRNAs, and sense lncRNAs (Additional File 1: Figs. S5B, S5C, S5D and S5E). We identified 251 lncRNA transcripts that were upregulated (Additional File 2: Data S5) in the pronotum cuticle of the MR strain, and 321 that were downregulated in the same sample (Additional File 1: Fig. S5F). However, only 12 upregulated and eight downregulated lncRNA transcripts satisfied the condition FPKM > 10 (Additional File 1: Fig. S5G). Among these candidates, only *lnc19419.6*, *lnc19419.16* and *lnc19419.19* showed large FPKM values indicating a potential role in malathion resistance (Additional File 1: Fig. S5H). However, the predicted target genes of these three lncRNAs were not genes which up-regulated in pronotum between MR and MS strain (Additional File 2: Data S4 and Data S6). Further investigation revealed that the three *lnc19419* transcripts were too similar to distinguish, and we therefore used the conserved regions for expression analysis and functional profiling *in toto* (Additional File 1: Fig. S5I).

**Fig. 4:**
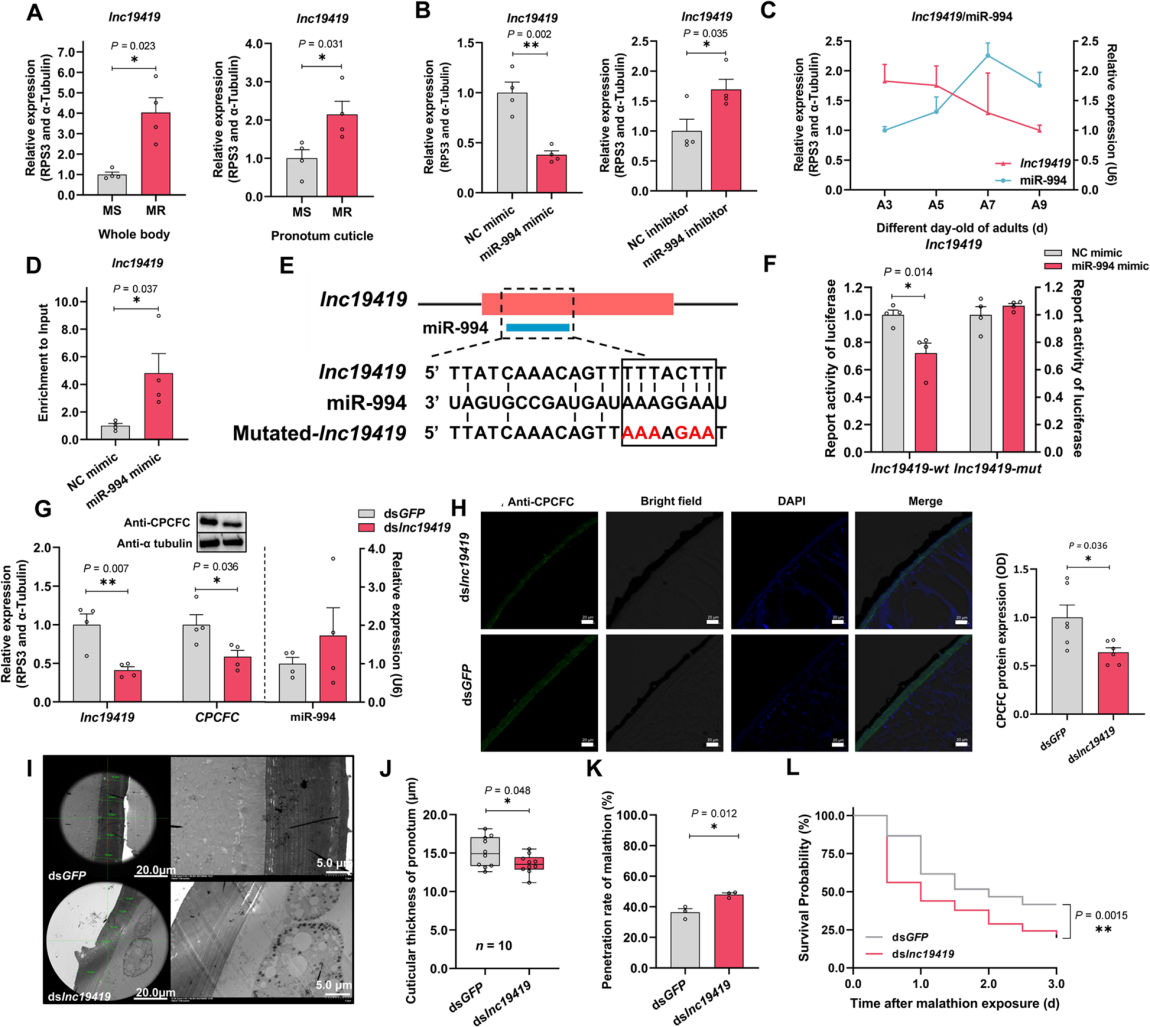
*lnc19419* regulate *CPCFC* which mediated malathion penetration resistance by sequestering miR-994. **A** Differential expression of *lnc19419* in the whole body and pronotum cuticle between the MR and MS strains. Each sample of whole body and pronotum cuticle contained four flies and 20 dissected pronotum of 5-day-old adult, respectively (*n* = 4). **B** Expression of *lnc19419* after injecting synthetic miR-994 or an inhibitor (*n* = 4). **C** Expression profiles of miR-994 and *lnc19419* in adult flies at 3, 5, 7 and 9 days old (A3, A5, A7 and A9, *n* = 4). **D** Enrichment analysis (biotin–avidin RNA pull-down) following the binding of miR-994 to *lnc19419* (*n* = 4). **E** Target site analysis, showing the interaction between miR-994 and *lnc19419*. Mutated-*lnc19419* contained the sequence 5′-AAAAGAA-3′ (red). **F** Luciferase activity indicating the targeting of miR-994 by *lnc19419 *in vitro. *lnc19419*-*wt* and *lnc19419*-*mut* indicate HEK293-T cells containing the 3′ UTR of wild-type and mutated *lnc19419*, respectively (*n* = 4). **G** *CPCFC* and *lnc19419* expression levels after silencing *lnc19419*.(*n* = 4). **H** The relative CPCFC content of pronotum cuticular sections after silencing *lnc19419*. CPCFC protein expression was determined by the fluorescence signal intensity over length in each sample (*n* = 6). **I** Analysis of pronotum cuticular ultrastructure after silencing *lnc19419*. **J** Analysis of the cuticular thickness after silencing *lnc19419* (*n* = 10). Each thickness value is the mean of five randomly selected cross-sectional measurements. **K** The malathion penetration rate after silencing *lnc19419.* Each eluant contained 30 5-day-old adult flies (*n* = 3). **L** The effect of *lnc19419* silencing on susceptibility to malathion. Survival rate was observed at 0–3 day after malathion exposure (*n* = 126)

To determine whether *lnc19419* was able to sequester miR-994, we used four prediction programs to determine miR-994 target sites in the 12 upregulate lncRNA transcripts. Eight lncRNA transcripts were selected by conjoint analysis involving all four predication programs (Additional File 1: Fig. S5J). Further analysis of 10 lncRNAs (*lnc19419.6*, *lnc19419.16* and *lnc19419.19* were uniformly analyzed as *lnc19419*) revealed that only *lnc19419* was regulated by overexpressing or inhibiting miR-994 (Fig. 4B and Additional File 1: Fig. S5K). The expression profiles of *lnc19419* and miR-994 were found to be complementary in adult flies at different ages (Fig. 4C). Based on these results, we hypothesized that *lnc19419* indirectly upregulates *CPCFC* in the pronotum cuticle of the MR strain by acting as a decoy for miR-994 (competitive sponging).

The relationship between *lnc19419* and miR-994 was confirmed in biotin–avidin RNA pull-down experiments. We observed a significant 5.8-fold enrichment of *lnc19419* compared to the negative control (Fig. 4D and Additional File 1: Fig. S5L). Furthermore, relative luciferase activity was significantly reduced by 27.9% when the plasmid pmirGLO-*lnc19419* (containing 5′-TTTACTT-3′) and synthetic miR-994 were co-transfected into HEK293T cells. In contrast, relative luciferase activity did not change when the plasmid contained mutated *lnc19419* (5′-AAAAGAA-3′) under the same conditions (Figs. 4E and F). In addition, in situ hybridization showed that the miR-994^Cy3^ and *lnc19419*^Fam^ were co-enriched in the pronotum cuticle (Additional File 1: Fig. S5M). These data confirm that *lnc19419* directly negatively regulates miR-994 in the pronotum cuticle of *B. dorsalis*.

The expression level of *lnc19419* was 3.0-fold higher in the whole body of MR flies compared to the MS strain, and 2.1-fold higher in the pronotum cuticle (Fig. 4A). To determine the relationship between *lnc19419* and *CPCFC*, *lnc19419* was efficiently knocked down (58.9%) in the MR strain by RNAi (Fig. 4G), in turn reducing *CPCFC* expression by 41.4% (Fig. 4G and Additional File 1: Fig. S4D). While silencing *CPCFC* also resulted in 60.6% reduction of *lnc19419* (Additional File 1: Fig. S5N). Immunohistochemical staining revealed that the CPCFC signal intensity was reduced by 36.1% in the pronotum cuticle (Fig. 4H). These data provide convincing evidence that *lnc19419* influences the CPCFC content of the pronotum cuticle. Accordingly, TEM analysis of the cuticle ultrastructure revealed significant thinning with 10.4% following the injection of double-stranded *lnc19419* RNA (Figs. 4I and J). The thinner cuticle led to an 11.6% increase in malathion penetration (Fig. 4K) and a 22.1% increase in malathion susceptibility post 72 h (Fig. 4L). These results were consistent with the data for miR-994 and *CPCFC*, and confirmed that *lnc19419* contributes to malathion resistance in *B. dorsalis* by modulating the cuticle thickness and thus the efficiency of malathion penetration.

### *lnc19419* ~ miR-994 ~ *CPCFC* regulatory axis in a field strain of *B. dorsalis*

**Fig. 5:**
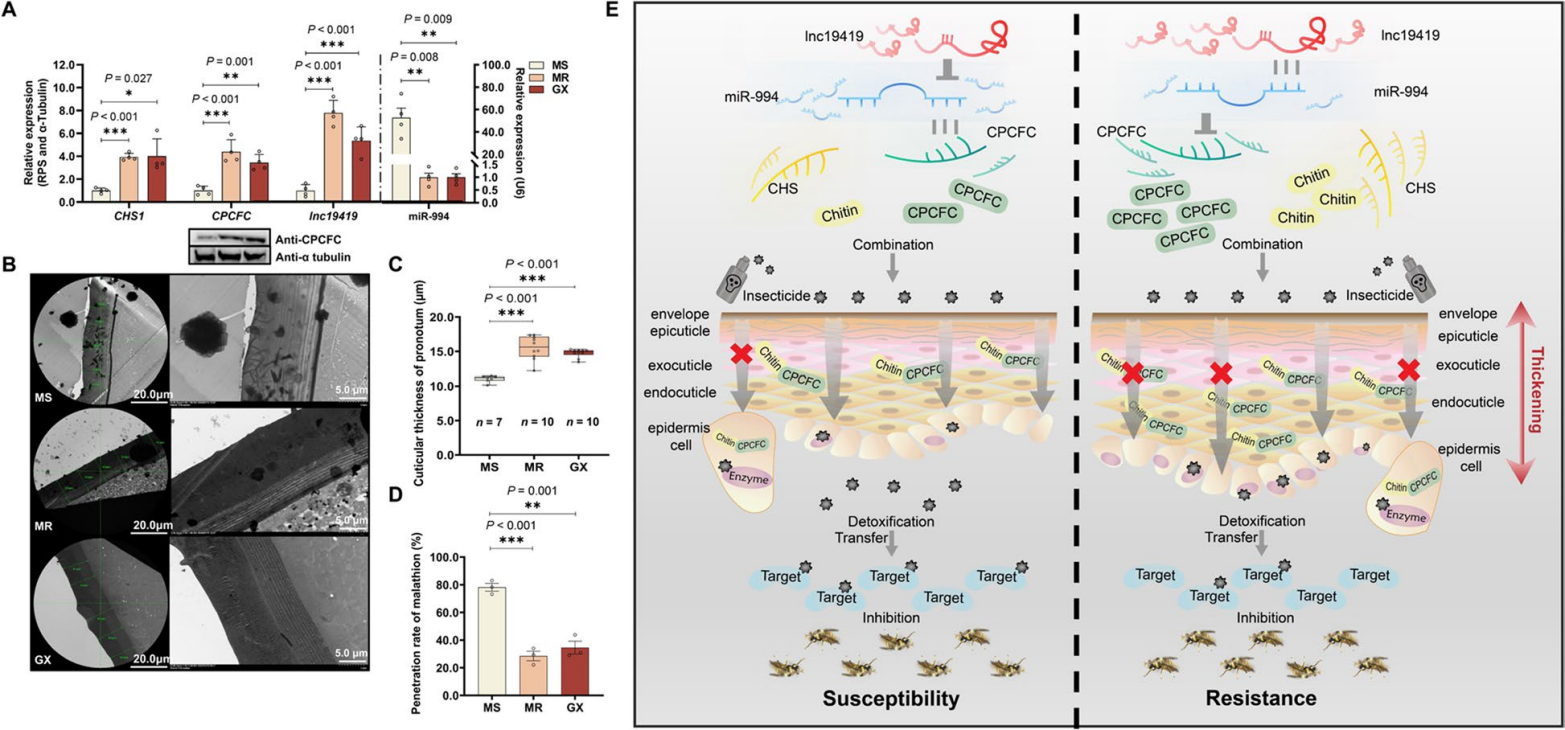
Proposed model of the *lnc19419* ~ miR-994 ~ *CPCFC* regulatory cascade that mediates insecticide penetration resistance in *B. dorsalis*. **A** Expression of *CHS1*, *CPCFC*, *lnc19419* and miR-994 in the MS, MR and GX strains (*n* = 4). **B** The cuticular ultrastructure of the MS, MR and GX strains. **C** The cuticular thickness of the MS (*n* = 7), MR (*n* = 10) and GX (*n* = 10) strains. Each thickness value is the mean of five randomly selected cross-sectional measurements. **D** Malathion penetration rate in the MS, MR and GX strains. Each eluant of MR, GX and MS strains contained 30, 50 and 50 5-day-old adult flies, respectively (*n* = 3). **E** In the MR strain, abundant *lnc19419* sequesters miR-994 by competitive sponging and weakens the ability of miR-994 to suppress *CPCFC*. The upregulation of *CPCFC* and *CHS1* in the MR strain provides abundant chitin and cuticular proteins for cuticular thickening, resulting in insecticide penetration resistance. The overexpression of detoxification enzymes in the epidermis cells of the MR strain enhances insecticide resistance even further by metabolizing the smaller amount of insecticide that does cross the barrier

The *lnc19419* ~ miR-994 ~ *CPCFC* regulatory axis not only regulates resistance in a laboratory strain (MR) of *B. dorsalis*, but was also found in a Guangxi (GX) field strain. The expression of miR-994 was downregulated by 98.1%, but *CHS1*, *CPCFC* and *lnc19419* were upregulated by 4.01-, 3.44- and 5.37-fold respectively in the GX strain compared to MS *(*Fig. 5A and Additional File 1: Fig. S4F). Similarly, the cuticle was thicker (1.33-fold) and the malathion penetration ratio was lower (43.6%) in the GX strain (Figs. 5B, C and D). The GX strain is resistant to four different classes of insecticides albeit at a low level, with the strongest resistance against chlorpyrifos (Additional File 3: Table S2). To investigate the cuticle-mediated insecticide resistance of *B. dorsalis* in the field and verify the laboratory data, we injected malathion and chlorpyrifos into the GX strain, resulting in a much higher susceptibility with 2.63- and 1.65- fold to both compounds respectively (Table 2). Taken together, these results might contribute to reveal and understand the resistance mechanism of *B. dorsalis* in field.

**Table 2:** Toxicity of malathion and chlorpyrifos against *B. dorsalis* of GX strain

Delivery method	Insecticides	LD_50_ (95% ng/fly)	Y = LD – P line	χ^2^	Topical/ injected ratio
**Topical application**	malathion	170.76 (153.63–189.37)	Y = 3.24X – 7.23	2.98	-
	chlorpyrifos	37.00 (33.12–41.56)	Y = 3.27X – 5.13	3.67	-
**Injection**	malathion	64.71 (48.38–92.20)	Y = 2.84X – 5.15	5.77	2.64^a^
	chlorpyrifos	22.40 (20.19–24.44)	Y = 3.87X – 5.22	2.69	1.65^b^

^a^LD_50_ of malathion topical application/injection

^b^LD_50_ of chlorpyrifos topical application/injection

The combined results set out above confirmed our hypothesis that insecticide penetration resistance in the pronotum cuticle of *B. dorsalis* is modulated by a regulatory cascade involving lncRNA, miRNA and mRNA. Briefly, the abundant *lnc19419* acts as a competitive sponge to sequester miR-994, preventing its negative interaction with *CPCFC* mRNA and increasing CPCFC expression (Fig. 5E). CPCFC thus accumulates in the cuticle and interacts with the abundant chitin synthesized by the upregulated *CHS1* in the MR strain. The CPCFC-chitin complex thickens the pronotum cuticle, creating a more effective barrier against insecticide penetration and thus increasing malathion resistance (Fig. 5E).

## Discussion

Chemical pesticides play a key role in the control of insect pests, while irrational application of pesticides pose a severe threat to human health and food security [27]. The effectiveness of chemical control is compromised by the ability of insects to evolve resistance to insecticides [28], a phenomenon that is widespread and rapidly increasing in spectrum and intensity [29]. Many resistance mechanisms involve genetic changes which facilitate insecticide detoxification (metabolic resistance) or alter the structure of insecticide targets (target site resistance), but additional mechanisms include avoidance behavior and the strengthening of barriers (penetration resistance) [3, 30]. The analysis of these additional mechanisms could reveal how insects evolve the most striking resistance phenotypes.

Most research on penetration resistance to date has focused on mosquitoes [6, 31], especially the mechanisms that result in cuticular thickening [3]. We confirmed the role of the cuticle thickening in penetration-resistant *B. dorsalis* by silencing *CHS1* and by comparing different delivery methods for the insecticide malathion. More than 43.6% of the resistance was thus eliminated by blocking the function of the cuticle with direct injection of insecticide, indicating the important role of cuticle in insecticide resistance of *B. dorsalis*. Penetration resistance could be mediated by cuticular lipids or hydrocarbons, but *CYP4G* (the key enzyme involved in lipid and hydrocarbon synthesis) is consistently expressed in the MR and MS strains [32, 33]. Furthermore, 20 genes encoding cuticle proteins were upregulated in the pronotum of the MR strain (based on RNA-Seq analysis), indicating that cuticular structure and thickness are associated with insecticide resistance. In addition, the RNA-Seq data also revealed the upregulation of some detoxification enzymes in the MR strain, which could possibly explain the higher resistance ratio even when malathion was injected and therefore bypassed the cuticle. This analysis indirectly supported the present and previous studies that penetration resistance in combination with other mechanisms could lead to much higher resistance levels in insect pests [25, 32].

To analyze the regulatory mechanisms of the putative penetration resistance, the small RNA-Seq was performed for MR and MS strains. We found that the miR-994 had high expression levels and it was the only one out of the four miRNAs identified that was down-regulated in the pronotum cuticle of the MR strain, suggesting a relationship between miR-994 and insecticide resistance. Subsequently, the evidence was confirmed that miR-994 modulated the penetration of the insecticide to increase susceptibility. This was supported by our finding that malathion penetration significantly increased following the introduction of synthetic miR-994. Indeed, the injection of synthetic miR-994 had a similar effect to silencing *CHS1*.

Given that miR-994 appears to control cuticular development, we investigated the effect of synthetic miR-994 on oriental fruit fly by transcriptome and compared the transcriptome of the pronotum integument between MR and MS strain. A substantial enrichment of the *CPCFC* mRNA (FPKM > 5,000) in the integument of the pronotum was discovered. CPCFC was first identified in nymphs of the death’s head cockroach (*Blaberus craniifer*) and usually contains three conserved motifs in which two cysteines are separated by five amino acids (C-X5-C) [34, 35]. Most insects only feature a maximum of two members of this unique cuticle protein family [36]. CPCFC was previously reported to influence cuticular development and insecticide resistance although the mechanism was not described [7, 35]. Interestingly, *CPCFC* is immediately up-regulated following ecdysis in the mosquito *Anopheles gambiae* [35], which is in contrast to the strong expression of most cuticle proteins before ecdysis [4, 35]. This indicates a unique role in cuticle development. We identified three lines of evidence demonstrating role of CPCFC in cuticle in *B. dorsalis*. First, immunohistochemical analysis revealed that CPCFC and chitin were co-located in cuticle pronotum. Second, the localization of CPCFC by immunogold labeling proved that it was secreted by epidermic cells and accumulates in the endocuticle of the pronotum. Third, recombinant CPCFC binds strongly to chitin *in vitro*. *CPCFC* was also strongly expressed in 1-day-old adults but expression declined with aging post-eclosion. Although it would be premature to conclude that CPCFC is involved the ecdysis, it clearly takes part in the cuticular development of *B. dorsalis* during post-eclosion.

Our cuticular RNA-Seq data comparing the MS and MR strains showed that *CPCFC* was expressed at the highest level among 20 differentially expressed cuticle protein genes. This suggests *CPCFC* is involved in cuticular development and is potentially responsible for cuticular thickening in the MR strain. Cuticular thickness was significantly reduced when *CPCFC* was targeted by RNAi, increasing the penetration of malathion and thereby the susceptibility of *B. dorsalis* to this insecticide. These data suggest that CPCFC may block the penetration of malathion via modulating cuticle, maintaining its in vivo concentration at a safe level for the MR strain because the smaller amount of malathion that does cross the barrier is more easily metabolized by detoxification enzymes.

Finally, the potential regulatory mechanisms of *lnc19419* were assessed via predication analysis. Although few studies have been reported in arthropods [19, 37], lncRNA genes are often linked to the genes they regulate [17]. We predicated the target mRNA of lncRNAs via searching adjacent genes in genome [38] of *B. dorsalis* like other species for lncRNA target analysis to analyze the potential regulatory mechanism of *lnc19419*. Total 12 potential adjacent genes of *lnc19419* was predicated including CPs, protein naked cuticle and kinase anchor protein, however the predicted target genes of *lnc19419* were not upregulated according to our RNA-Seq data comparing the MR and MS strains. It provides possibility that *lnc19419* might participate in insecticide resistance via another indirect regulatory mechanism. While the predication results of miR-994 revealed the potential lncRNAs target including *lnc19419*. Thus, we considered the possibility that miR-994 may be regulated by lncRNAs acting as decoys [39]. Further investigation by RNA pull-down and dual-luciferase reporter assays confirmed that miR-994 is negatively regulated by *lnc19419*. The *lnc19419* and miR-994 signals overlapped in the pronotum cuticle supporting their relationship in vivo. The expression of *CPCFC* was significantly reduced when we injected a double-stranded construct matching *lnc19419* resulting in a similar phenotype to the silencing of *CPCFC*, including a higher malathion penetration ratio, higher susceptibility to malathion, and a thinner pronotum cuticle. In addition, the regulatory axis of *lnc19419* ~ miR-994 ~ *CPCFC* was also confirmed in a field strain via expression investigation and might be responsible for the complex resistance background of GX strain, indicating the universality of the regulatory axis in *B. dorsalis*. Taken together, our data suggested that malathion resistance in *B. dorsalis* is mediated by a regulatory axis involving *lnc19419*, miR-994 and *CPCFC* mRNA.

## Conclusions

In conclusion, long-term selection revealed that the *B. dorsalis* integument is a critical tissue in the evolution of malathion resistance, which is mediated by cuticular thickening. The underlying mechanism is based on a regulatory axis of *lnc19419* ~ miR-994 ~ *CPCFC* expression in dynamic equilibrium, which favors the thickening of the cuticle in *B. dorsalis*. This regulatory axis blocks insecticides by increasing the penetration distance, reducing the entry amount of insecticide into insect. Our work provides insight into the efficacy of contact insecticides against insect pests and may facilitate the development of safe insecticides with greater specificity to agricultural pests and insect vectors of human diseases targeting endogenous RNAs.

## Methods

### Insects and insecticide bioassays

The malathion-susceptible (MS) strain was originally collected from Guangdong province, China, in 2009. It was never exposed to any insecticide and was reared in our laboratory. The malathion-resistant (MR) strain (Guangdong, China, 2008) was obtained by continuous selection with 89.1% malathion (Institute for Control of Agrochemicals, Sichuan, China) applied to the pronotum of adult flies using a PB600-1 repeating dispenser [26] (Hamilton Company, Reno, NV, USA) for 26 generations. In addition, a field strain of *B. dorsalis* (GX strain) was collected from Guangxi province, China, in 2021. These strains were maintained at 27 ± 1 °C and 70 ± 5% relative humidity with a 14-h photoperiod in the laboratory, and were fed on an artificial diet [40]. All experiments complied with ethical regulations for *B. dorsalis* testing and research.

For topical insecticide application, 5-day-old adult flies were cooled to –20 °C for 2 min before malathion was applied to the pronotum using a PB600-1 repeating dispenser (Additional file 1: Fig. S1A). For insecticide injection, various concentrations of insecticide were injected into adult flies via the thorax using a Nanoject II Auto-Nanoliter Injector (Drummond Scientific, Broomall, PA, USA) (Additional file 1: Fig. S1A). Approximately 20 flies were transferred to each cage, and three replicates were exposed to each concentration. Mortality was recorded over the next 72 h and data were analyzed using SPSS v22.0 for Windows (IBM, Chicago, IL, USA).

### Insecticide penetration analysis

Insecticide penetration was measured as previously described, with slight modifications [32, 41]. Flies were exposed to malathion at the LD_50_ for 1 h before washing twice with 3 mL acetonitrile in a 50-mL centrifuge tube. The eluent was then concentrated and dried in a CoolSafe (LaboGene, Bjarkesvej, Denmark) and dissolved in 1 mL acetonitrile. The solution was centrifuged (12,000* g*, 10 min, 4 °C) and the malathion content of the supernatant was determined by high performance liquid chromatography (HPLC, Agilent 1260 LC, Agilent Technologies, Palo Alto, CA, USA) as previously described [22, 42]. Briefly, the 10 μL sample was eluted with reversed-phase analytical column (ZORBAX SB-C18, 4.6 × 100 mm, 3.5 μm, Agilent Technologies) under mobile phase (60% acetonitrile and 40% ultrapure water) with speed of 1.0 mL·min^−1^. The malathion was analyzed with monitor at 27 °C and 230 nm. The penetration ratio of malathion was calculated as follows:${\mathrm{penetration}\;\mathrm{ratio}=\frac{\mathrm{total}\;\mathrm{applied}\;\mathrm{dose}\;-\;\mathrm{residual}\;\mathrm{malathion}}{\mathrm{total}\;\mathrm{applied}\;\mathrm{dose}}}\times100\%$.

### Chitin content analysis

The chitin was hardly extracted and measured from insects, thus the reaction production glucosamine reflected the content of chitin. It was measured as previously described, with slight modifications [5]. The flies were weighed, homogenized in PBS and transferred to 3% SDS. The mixture was heated to 100 °C for 15 min and centrifuged (1,800* g*, 10 min, 4 °C). We then added 300 μL 14 M KOH to the pellet and incubated at 130 °C for 90 min. The samples were then mixed with 800 μL ice-cold 75% ethanol and incubated on ice for 15 min before adding 30 μL Celite 545 (0.1 g in 2 mL 75% ethanol). After centrifugation (1,800* g*, 15 min, 4 °C), the pellet was washed with ice-cold 40% ethanol to purify the chitosan. We then mixed 500 μL chitosan with 250 μL 10% NaNO_2_ and 250 μL 10% KHSO_4_ and incubated at room temperature for 15 min. After centrifugation (1800* g*, 15 min, 4 °C), 120 μL of the supernatant was vortexed with 40 μL NH_4_SO_3_NH_2_ (2.5 g dissolved in 17.5 mL ultrapure water) for 15 min before adding 40 μL 3-methyl-2-benzothiazolone (MBTH) hydrazone hydrochloride hydrate solution (freshly prepared, 0.05 g dissolved in 10 mL ultrapure water) and incubating at 100 °C for 5 min. After cooling, the reaction mixture was measured with glucosamine at 650 nm using a microplate reader to analyze the chitin content.

### Cuticular ultrastructure and colloidal gold immunolocalization

The pronotum of 5-day-old adult flies was dissected, fixed in 4% glutaraldehyde at 4 °C overnight, washed with PBS and transferred to a tube containing 1% osmium tetroxide for 2 h. After another wash with PBS, the samples were dehydrated in a gradient of 60%, 70%, 80%, 90%, 95% and 100% ethanol (1 h each) followed by acetone for 1 h, before incubation in Suprr (Ted Pella, Inc., Redding, CA, USA) in a temperature gradient of 40, 50, 60 and 70 °C. Ultrathin sections (< 70 nm) were prepared on an ultramicrotome (Leica Microsystems, Frankfurt, Germany) and stained with 2% aqueous uranyl acetate for 10 min. After washing with ultrapure water, the dried ultrathin sections were imaged by TEM on an HT7800 instrument (Hitachi, Tokyo, Japan). For gold immunolocalization, the pronotum of 5-day-old adult flies was dissected, fixed in 3% paraformaldehyde and 0.5% glutaraldehyde at 4 °C overnight, wished with PBS and dehydrated with 30% and 50% ethanol at 4 °C (20 min each). Then, the samples were further dehydrated with 50%, 70%, 90% and 100% ethanol (40 min each) and treated with LR Gold (Ted Pella, Inc.) contained 30%, 70% and 100% ethanol (120 min each) at -20 °C. After that, the samples incubated with LR Gold resin under ultraviolet for 72 h. Then, ultrathin sections (< 70 nm) were prepared on an ultramicrotome and blocked with 5% goat serum for 1 h before incubation with an antibody specific for CPCFC (AtaGenix, Wuhan, China; diluted 1:100) at 4 °C overnight. After three washes in PBS, bound primary antibody was detected using a goat anti-rabbit IgG conjugated to 10-nm colloidal gold (Thermo Fisher Scientific, Waltham, MA, USA; diluted 1:20) at room temperature for 2 h. After washing with PBS and ultrapure water, the ultrathin section was stained with 2% aqueous uranyl acetate for 10 min prior to TEM.

### Quantitative real-time analysis of miRNA, lncRNA and mRNA

Each sample for RT-qPCR contained four 5-day-old adult flies. Total RNA was extracted from flies with TRIzol reagent. For mRNA and lncRNA, genomic DNA was removed with RQ-Free DNase (Promega, Madison, WI, USA) and first-strand cDNA was prepared using the PrimeScript 1st Strand cDNA Synthesis kit (TaKaRa, Dalian, China). Quantitative real-time analysis of mRNA and lncRNA was carried out as previously described with GoTaq qPCR Master Mix (Promega) [42, 43]. For miRNA, the cDNA was synthesized using the miRcute Plus miRNA First-Strand cDNA Kit and quantitative real-time analysis was carried out using the miRcute Plus miRNA qPCR Kit (both kits from Tiangen, Beijing, China) by CFX384 Touch™ (Bio-Rad, Hercules, CA, USA). The relative expression levels of miRNA, mRNA and lncRNA were analyzed using qBase^+^ (Additional File 3: Table S3) [44].

### Small RNA transcriptome sequencing

Total RNA was extracted from 5-day-old adult flies (MR and MS strains) using TRIzol reagent. We prepared three biological replicates, each a mixture of two female and two male flies. The purity, concentration and integrity of RNA were tested using a NanoDrop 2000 spectrophotometer (Thermo Fisher Scientific) and by gel electrophoresis to ensure the use of qualified samples for transcriptome sequencing. The six small RNA libraries from the MR and MS strains were prepared for the Illumina HisSeq 2500 platform by Biomarker Technology (Beijing, China). RNA-Seq and the identification of miRNAs followed our previous approach [45]. The differential expression of miRNAs between the MR and MS strains was determined using DESeq2 (*P* < 0.05 and |Fold change|> 1) [46].

### Biotin–avidin RNA pull-down

Biotin–avidin RNA pull-down experiments were carried out as previously described with slight modifications [37, 47]. The synthetic miR-994 and negative control (NC) conjugated to biotin were injected into 3-day-old MR flies. Eight injected flies per sample were homogenized in lysis buffer (20 mM Tris–HCl pH 7.5, 100 mM KCl, 5 mM MgCl_2_, 0.3% Triton X-100) containing RNAse inhibitor and protease inhibitor (Thermo Fisher Scientific). After centrifugation (18,000* g*, 10 min, 4 °C), the supernatant of each sample was divided into two parts (the input sample and pull-down sample). The pull-down sample was added to pre-treated magnetic beads (washed with solutions A and for five times), which contained avidin, for incubation at 4 °C overnight. After a centrifugal pulse, the supernatant was removed from each sample using a magnetic stand (Beyotime Biotechnology, Shanghai, China). Then, the beads were washed four times with lysis buffer. Total RNA was then extracted from the input sample and washed pull-down sample using TRIzol reagent, and first-strand cDNA was prepared as described above. The enrichment of mRNA/lncRNA was determined by RT-qPCR using *B. dorsalis ribosomal protein S3* (*RPS3*) and *α-tubulin* as reference genes (Additional File 3: Table S3) with four biological replicates.

### Co-localization analysis of RNA by in situ hybridization

The pronotum was dissected from 5-day-old adults (MS strain) and fixed in 4% paraformaldehyde at 4 °C overnight. Samples were then embedded in optimum cutting temperature (O.C.T.) compound (Sakura, Tokyo, Japan) at –80 °C for 2 h before 8 μm sections were prepared on a freezing microtome (Thermo Fisher Scientific). The target RNAs were detected using an in situ hybridization kit (Gefan Biotechnology, Shanghai, China). Probes complementary to miR-994, *lnc19419* and *CPCFC* mRNA were labeled with Cy3 or Fam (Gefan Biotechnology; Additional File 3: Table S3). Signals were visualized under a laser scanning confocal microscope (Carl Zeiss, Oberkochen, Germany).

### Analysis of CPCFC protein levels in vivo

CPCFC was detected in vivo by western blot and immunohistochemical analysis as previously described [6, 7]. For western blots, 10 flies were extracted in lysis buffer (50 mM Tris–HCl pH 7.5, 8 M urea, 0.2 M NaCl, 0.1% SDS) and using the BCA Protein Assay Kit (Beyotime Biotechnology) to quantify extracted protein. The polyclonal antibody of CPCFC and α-tubulin were respectively generated in rabbits with the synthetic recombinant peptide (AtaGenix) (Additional File 3: Table S3). The CPCFC and α-tubulin antibody were diluted by antibody diluent (Beyotime Biotechnology) at 1:1,000. For immunohistochemical analysis, 8 μm sections of the pronotum were prepared as described above, and imaged by LSCM. The CPCFC antibody was diluted by PBST (phosphate buffered saline pH7.5) with 1:200. The fluorescence intensity was analyzed using ZEN v2.3 (blue edition) software (Carl Zeiss).

### Luciferase reporter analysis

Luciferase reporter assays were carried out as previously described [48]. The *CPCFC* mRNA sequence including the 3′UTR and the *lnc19419* sequence were each inserted into the vector pmirGLO (Promega). Mutated versions were prepared using a site-directed mutagenesis kit (Beyotime Biotechnology). The four plasmids were then used in separate transfection experiments, and were introduced into HEK-293 T cells along with synthetic miRNAs using transIt-LT1 transfection reagent (Mirus Bio, Madison, WI, USA). The relative luciferase activity was measured 24 h post-transfection with an Orion microplate luminometer (Berthold Technologies, Bad Wildbad, Germany) and the dual-glo luciferase assay system (Promega).

### Analysis of CPCFC binding to chitin

The chitin-biding ability of CPCFC was tested in a bacterial expression system [42]. The *CPCFC* open reading frame was inserted into vector pET-28b and expressed in *Escherichia coli* BL21(DE3) cells (Biomed, Beijing, China). The recombinant protein was purified using a Ni–NTA spin column (Sangon, Shanghai, China) and correct folding was confirmed by western blot. The soluble recombinant CPCFC was then mixed with chitin resin (Sigma-Aldrich) and incubated at room temperature for 4 h [49]. After centrifugation (12,000* g*, 10 min, 4 °C) the supernatant and pellet were collected to analyze the CPCFC content by western blot. The protein content of the supernatant was also measured using a BCA protein assay kit (Beyotime Biotechnology).

### Transcriptome sequencing (mRNA and lncRNA)

RNA libraries were prepared for the Illumina HiSeq platform (Biomarker Technology) and were sequenced and analyzed as previously described [43]. In brief, the total RNA of miR-994 mimic treatment and pronotum of both MR and MS strains were extracted with Trizol reagent and RNA libraries were constructed (Biomarker Technology). The rRNA was wiped off from the total RNA using Epicentre Ribo-Zero™ kit (Epicentre, Madison, WI, USA). The rRNA-depleted RNA was disturbed randomly and synthesized with random hexamers. The purified double stranded cDNA was repaired at the ends, and the adaptor-ligated was added and sequenced. Then AMPure XP beads were used to select the size of the fragment and degraded the cDNA that contained UDGs. After enriching the cDNA library and examined the quality of library, the sequencing was performed on the platforms of Illumina HiSeq™ (Biomarker Technology). The raw data of reads were purified into clean reads and mapped to the genome of *B. dorsalis* (GCF_000789215.1, ASM78921v2.*Bactrocera_dorsalis*.ASM78921v2.genome.fa) and related annotation files (GCF_000789215.1_ASM78921v2_genomic.gff, https://www.ncbi.nlm.nih.gov/genome/?term=bactrocera+dorsalis) [50] using HISAT2 [51]. The final transcriptome was assembled and the FPKM value was calculated using StringTie [52].

The analysis and identification of lncRNA was performed as our previous studies [16, 43]. The class-code of transcripts with ‘i’, ‘x’, ‘u’, ‘o’ and ‘e’ were chosen and screened out the transcripts with length > 200 bp, exon numbers > 2 and FPKM value > 0.1. In addition, the transcript was evaluated whether it possessed the capability of coding protein using CPC (Coding Potential Calculator, score < 0) [53], CNCI (Coding-Non-Coding Index, score < 0) [54], CPAT (Coding-Potential Assessment Tool, identified label was ‘nocoding’) [55] and Pfam (E-value < 0.001) [56] analysis. The rest of the transcripts were classified into four types of lncRNA via Cuffcompare [57].

For the analysis of differential expression post injecting synthetic miR-994 or NC, total RNA was extracted with Trizol reagent from three replicate samples of two males and two females injected with the synthetic miR-994 or NC. For cuticular RNA-Seq (MR and MS strains), 20 dissected pronotum without muscle from 5-day-old adult flies (10 male and 10 female) were pooled as a single sample. Three replicate samples were prepared from each strain. The differential expression of mRNA and lncRNA was analyzed using DESeq2 (*P* < 0.05 and |Fold change|> 1) [46]. The lncRNA target genes were predicated by searching adjacent genes within a 100-kb range.

### Prediction of miR-994 target genes

Twenty genes were selected based on the overlap between the genes downregulated by the injection of synthetic miR-994 and the genes upregulated in MR flies in the cuticular RNA-Seq experiments. We also selected 12 lncRNAs upregulated in the cuticular RNA-Seq experiments that exceeded a FPKM threshold of 10. These sequences were analyzed using miRanda [58], PITA [59], RNAhybrid [60], and qTar (https://github.com/zhuqianhua/qTar) to predict miR-994 binding sites.

### RNAi and synthetic miR-994/inhibitor injection

The dsRNA of *CHS1*, *CPCFC*, *lnc19419* and *GFP* were synthesized using the transcript aid T7 high yield kit (Thermo Fisher Scientific). The dsRNA (2 μg) or synthetic miRNA (50 pmol, miR-994 or NC) were injected into 3-day-old adults of the MR strain, and a second injection was carried out 24 h later. The functional analysis of *lnc19419*, miR-994, *CHS1* and *CPCFC* was carried out 12 h after the second injection.

### Statistical analysis

Data are shown as means ± SE from at least three independent biological replicates. All Figures were prepared using GraphPad Prism v8.0 (GraphPad Software, San Diego, CA, USA). Statistical analysis was carried out using SPSS v22.0 for Windows. The Survival rate was analyzed with Log-rank (Mantel-Cox) test of GraphPad Prism v8.0 (*P < 0.05, **P < 0.01, ***P < 0.001). ﻿Means were compared using two-tailed Student’s *t*-tests with significance levels set at *P < 0.05, **P < 0.01 and ***P < 0.001 (ns, no significant difference, *P* > 0.05). For cuticular thickness analysis, the boxes represent the 25% and 75% percentiles and the black lines within the boxes indicate the medians. Error bars correspond to the minimum and maximum values.

### Supplementary Information

**Additional file 1: Figures S1-S5. FigS1.** The cuticle-mediated resistance analysis. **FigS2. **Differential expression of small RNAs between the MR and MS strains.** FigS3. **Prediction of miR-994 target genes. **FigS4.** Western blot and immunohistochemical analysis.** FigS5. **The identification of lncRNAs and subsequent bioinformatics analysis.

**Additional file 2: Data S1-S6. Data S1. **Identified miRNAs and other sequences from small RNA-Seq experiments. **Data S2.** Downregulated miRNAs from small RNA-Seq experiments of MR strain. **Data S3.** Downregulated mRNAs following treatment with synthetic miR-994. **Data S4.** Upregulated mRNAs based on cuticular RNA-Seq experiments. **Data S5.** Upregulated lncRNAs based on cuticular RNA-Seq experiments.** Data S6. **Target genes predication for lncRNA.

**Additional file 3: Tables S1-S3. Table S1.** The analysis of detoxification enzyme activities following the injection of synthetic miR-994. **Table S2. **Toxicity of different insecticides against *B. dorsalis* strains MS and GX. **Table S3.** The information of primers, probe and antibody used in this study.

**Additional file 4. **The individual data values for Fig. 1A-C, E-G, 2A-C, 2E-H, 2J, 3A-B, 3D-F, 3H-J, 4A-D, 4E-H, 4J-L, 5A, 5C-D, S1B-C, S2A-D, S3C, S3E, S3G, S5G-H, S5K-L, S5N, Table. 1, 2, S1, S2.

## Data Availability

The RNA-Seq raw data of mimic injecting and pronotum cuticle are deposited in NCBI database with accession numbers PRJNA905490 [61] and PRJNA905195 [62]. miRNA-seq data presented in this article are deposited in NCBI database with accession number PRJNA905573 [63]. All data generated or analyzed during this study are included in this published article and its supplementary information files in Additional file 4. The other data supporting the findings of this study are available from the corresponding authors on request.
