# Supplementary material for: Cuticular competing endogenous RNAs regulate insecticide penetration and resistance in a major agricultural pest
Source: BMC Biol. 2023 Sep 5;21:187. doi: 10.1186/s12915-023-01694-z (PMC10478477; doi:10.1186/s12915-023-01694-z)
Supplement: Supplementary file 1 — Additional file 1: Figures S1-S5. FigS1. The cuticle-mediated resistance analysis. FigS2. Differential expression of small RNAs between the MR and MS strains. FigS3. Prediction of miR-994 target genes. FigS4. Western blot and immunohistochemical analysis. FigS5. The identification of lncRNAs and subsequent bioinformatics analysis. [file 12915_2023_1694_MOESM1_ESM.docx]

**Additional File 1: Figures S1-S5. FigS1.** The cuticle-mediated resistance analysis. **FigS2.** Differential expression of small RNAs between the MR and MS strains. **FigS3.** Prediction of miR-994 target genes. **FigS4.** Western blot and immunohistochemical analysis. **FigS5.** The identification of lncRNAs and subsequent bioinformatics analysis.


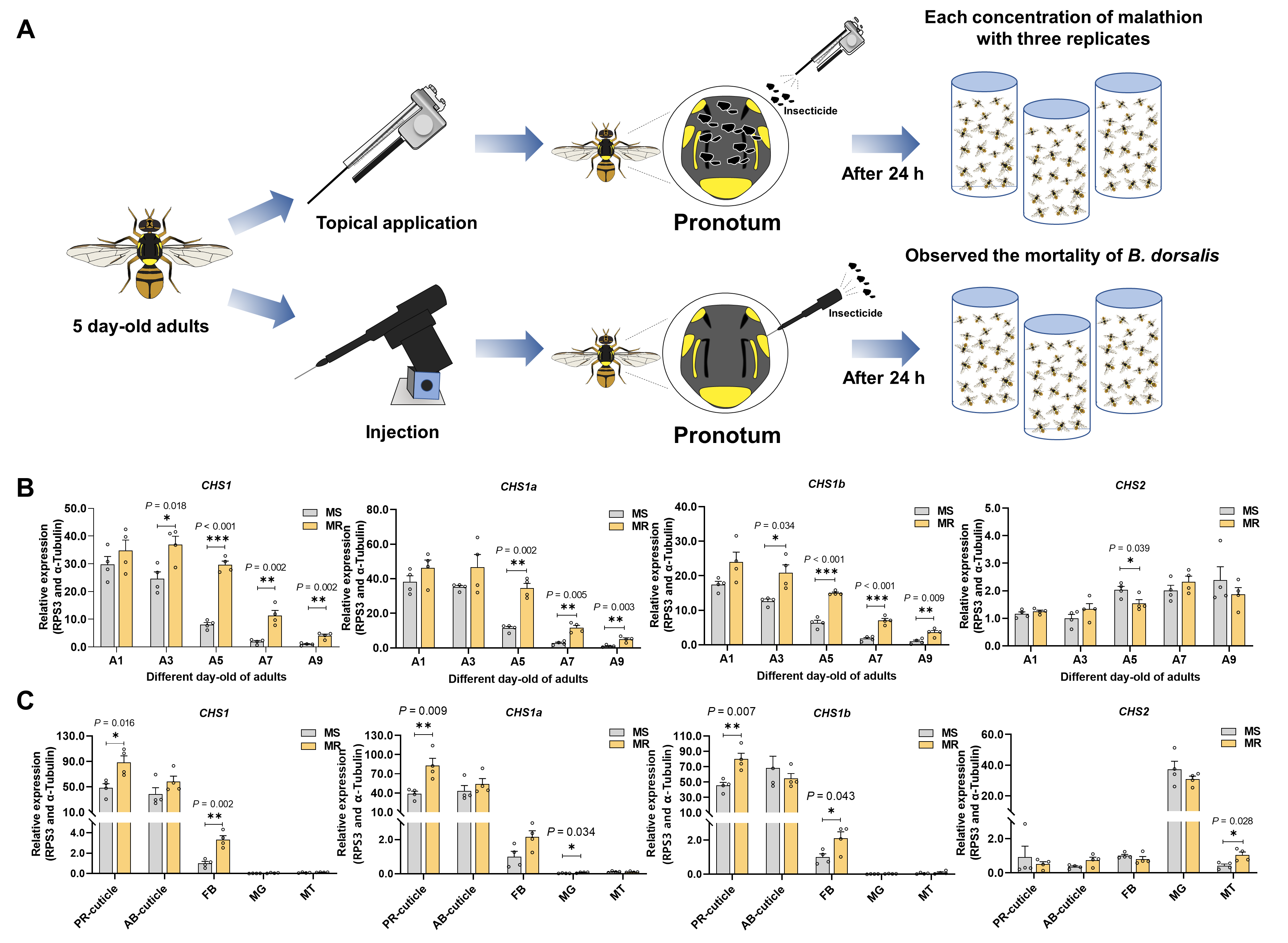


**Fig. S1. The cuticle-mediated resistance analysis.** (A) Bioassay to test the effect of malathion against *B. dorsalis* by topical application and injection. (B) The expression profiles of *CHS1*, *CHS1a*, *CHS1b* and *CHS2* in adult flies at different ages (MR and MS strains). Real-time quantitative PCR data are means ± SEM (*n* = 4). (C) The expression level of *CHS1*, *CHS1a*, *CHS1b* and *CHS2* in different tissues of 5-day-old of adults (MR and MS strains). Real-time quantitative PCR data are means ± SEM (*n* = 4). Differences in proportion were compared using Student’s *t*-test (**P* < 0.05, ***P* < 0.01, ****P* < 0.001).


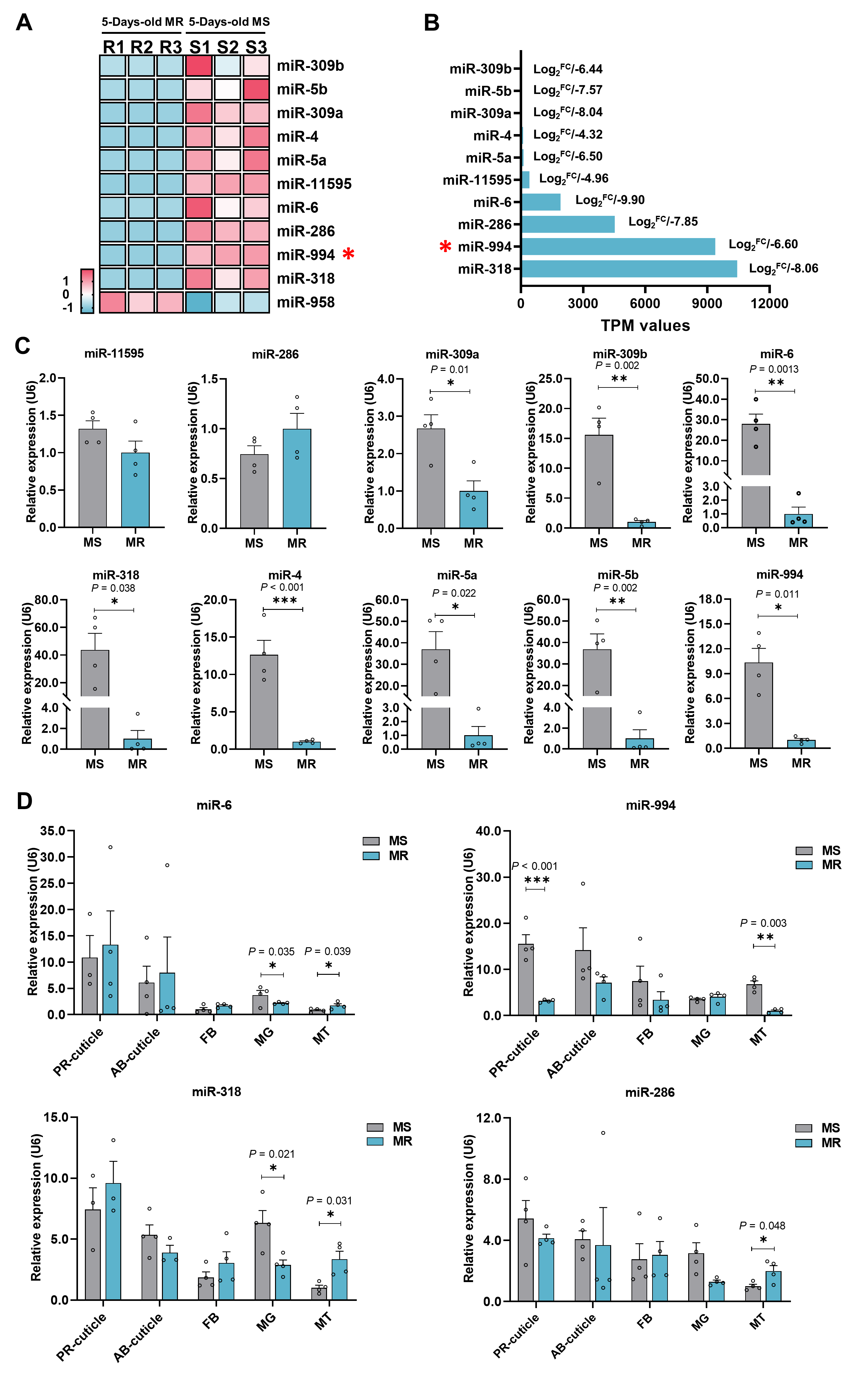


**Fig. S2. Differential expression of small RNAs between the MR and MS strains.** (A) Cluster values were analyzed with z-scores (from −1 to 1). (B) FPKM values of downregulated miRNAs. FPKM values are means of three biological replicates from the MS strain. (C) Real-time quantitative PCR data for the expression of miRNAs are means ± SEM (*n* = 4). (D) Expression profiles of four miRNAs with high FPKM values from different tissues of the MR and MS strains. Real-time quantitative PCR data are means ± SEM (*n* = 4). Each sample contained 20 fly-tissues of 5-day-old adult. Differences in proportion were compared using Student’s *t*-test (**P* < 0.05, ***P* < 0.01, ****P* < 0.001).


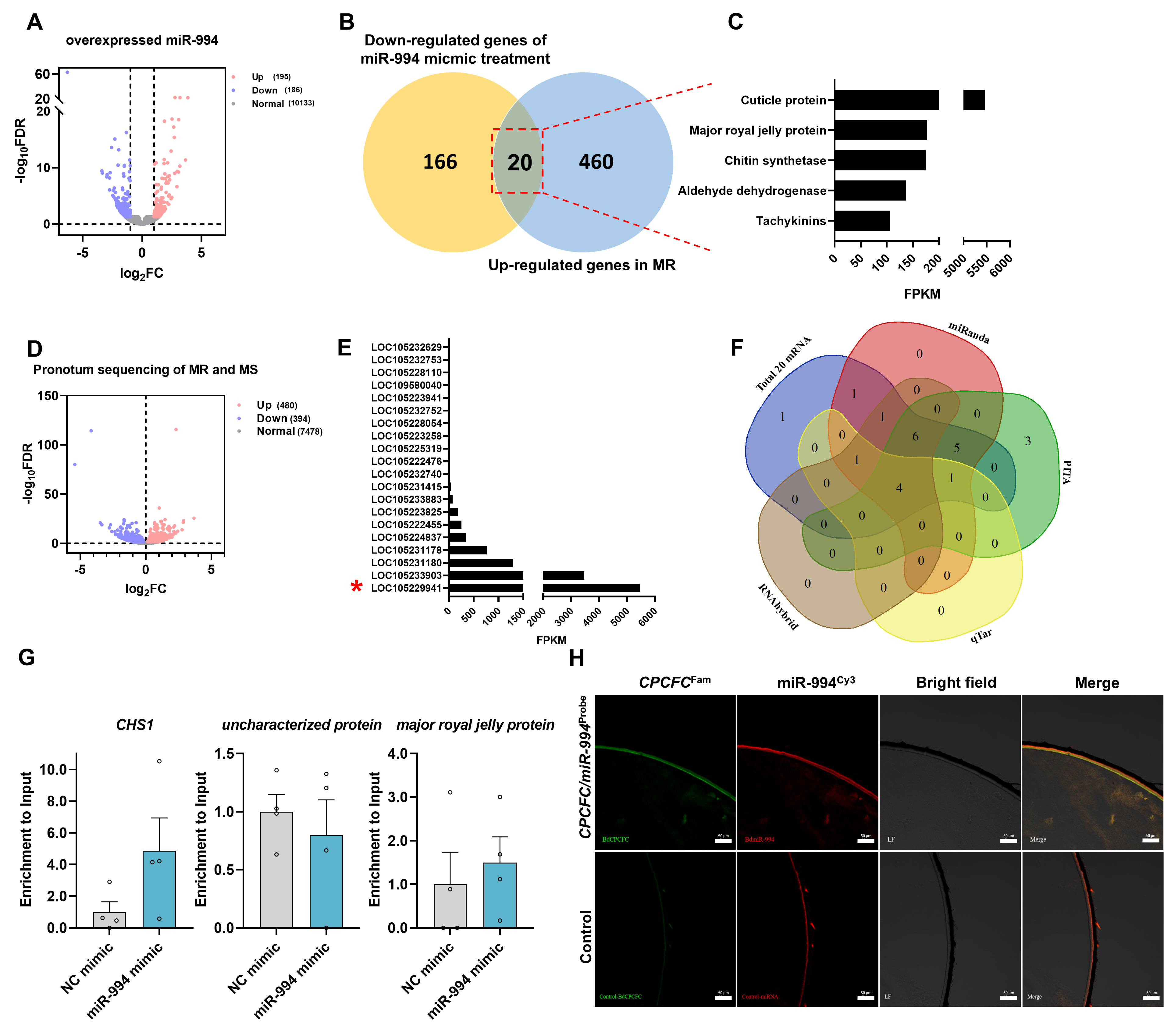


**Fig. S3. Prediction of miR-994 target genes.** (*A*) RNA-Seq data following the injection of synthetic miR-994. Differentially expressed genes related to the cuticle were identified by NR annotation. (*B*) Venn diagram showing the overlap between genes downregulated by synthetic miR-994 treatment and genes upregulated in the cuticle of MR flies. (*C*) FPKM values for top-five of the 20 overlapping genes. (*D*) Cuticular RNA-Seq data comparing the MR and MS strains. (*E*) FPKM values of 20 upregulated genes encoding cuticular proteins in the pronotum of the MR strain. The red asterisk indicates the *CPCFC* gene. (*F*) Analysis of the 20 overlapping genes using four algorithms that predict miRNA targets (RNA hybrid, qTar, PITA, and miRanda). (G) Enrichment analysis in the biotin–streptavidin RNA pull-down assay for the binding of miR-994 to potential target genes other than *CPCFC*. The three additional target genes were analyzed to determine their interactions *in vivo*. Each sample contained eight 5-day-old adult flies. Data are means ± SEM (*n* = 4). Differences in proportion were compared using Student’s *t*-test (**P* < 0.05, ***P* < 0.01, ****P* < 0.001). (H) Co-localization of miR-994 and *CPCFC* mRNA in the pronotum cuticle of 5-day-old adults. The green and red signals (Fam and Cy3 labeling) indicate *CPCFC* mRNA and miR-994, respectively.


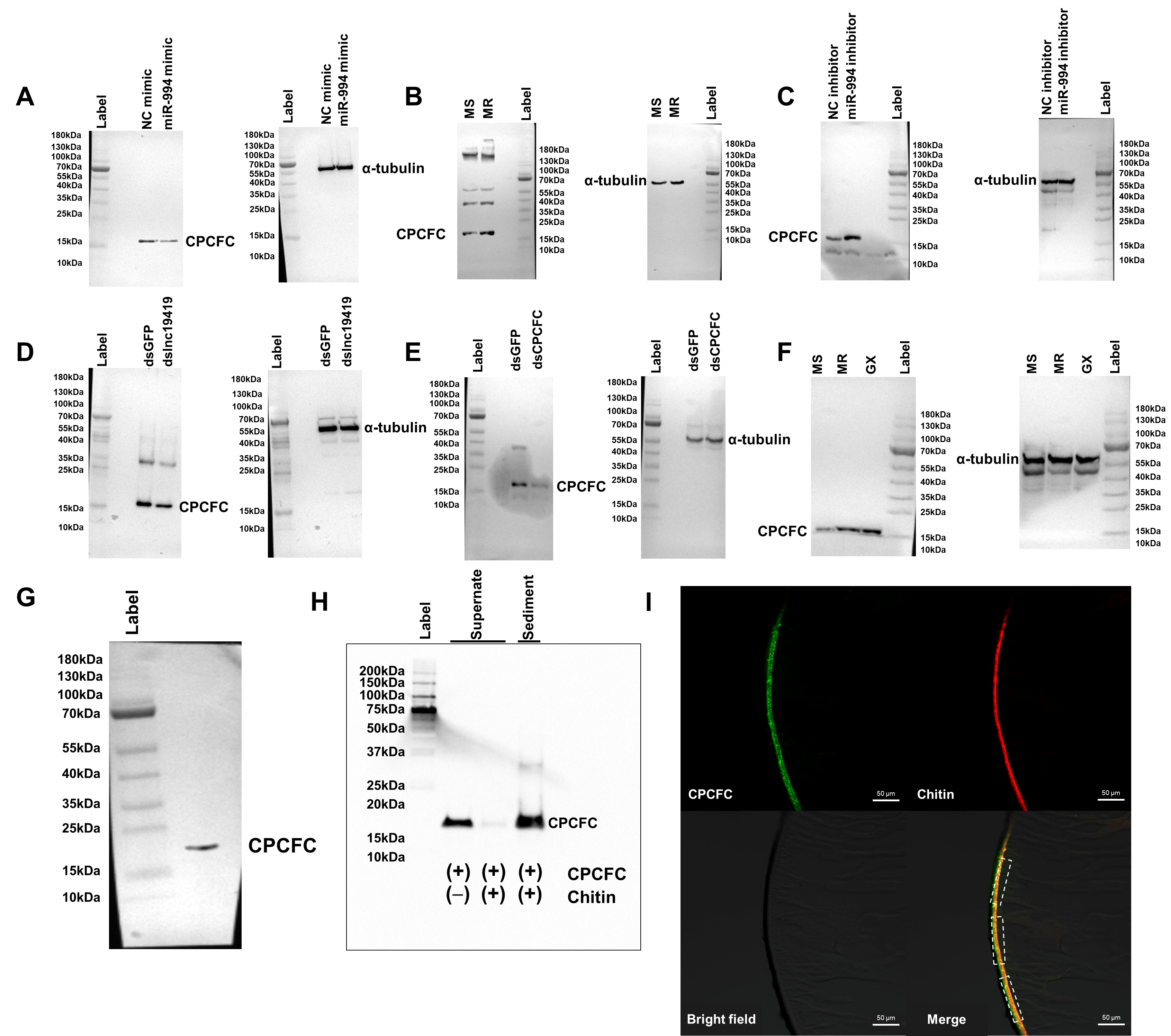


**Fig. S4. Western blot and immunohistochemical analysis.** (A) The CPCFC and α-tubulin content analysis post injecting synthetic miR-994 mimic. (B) The CPCFC and α-tubulin content analysis between MR and MS strain. (C) The CPCFC and α-tubulin content analysis post injecting synthetic miR-994 inhibitor. (D) The CPCFC and α-tubulin content analysis post silencing *lnc19419*. (E) The CPCFC and α-tubulin content analysis post silencing *CPCFC*. (F) The CPCFC and α-tubulin content analysis between MR, MS and GX strain. (G) The detection of recombinant CPCFC. (H) Chitin-binding analysis of recombinant CPCFC. (I) Immunohistochemical co-localization analysis of chitin and CPCFC in a section of pronotum cuticle. The green and red fluorescence signals indicate CPCFC and chitin, respectively.


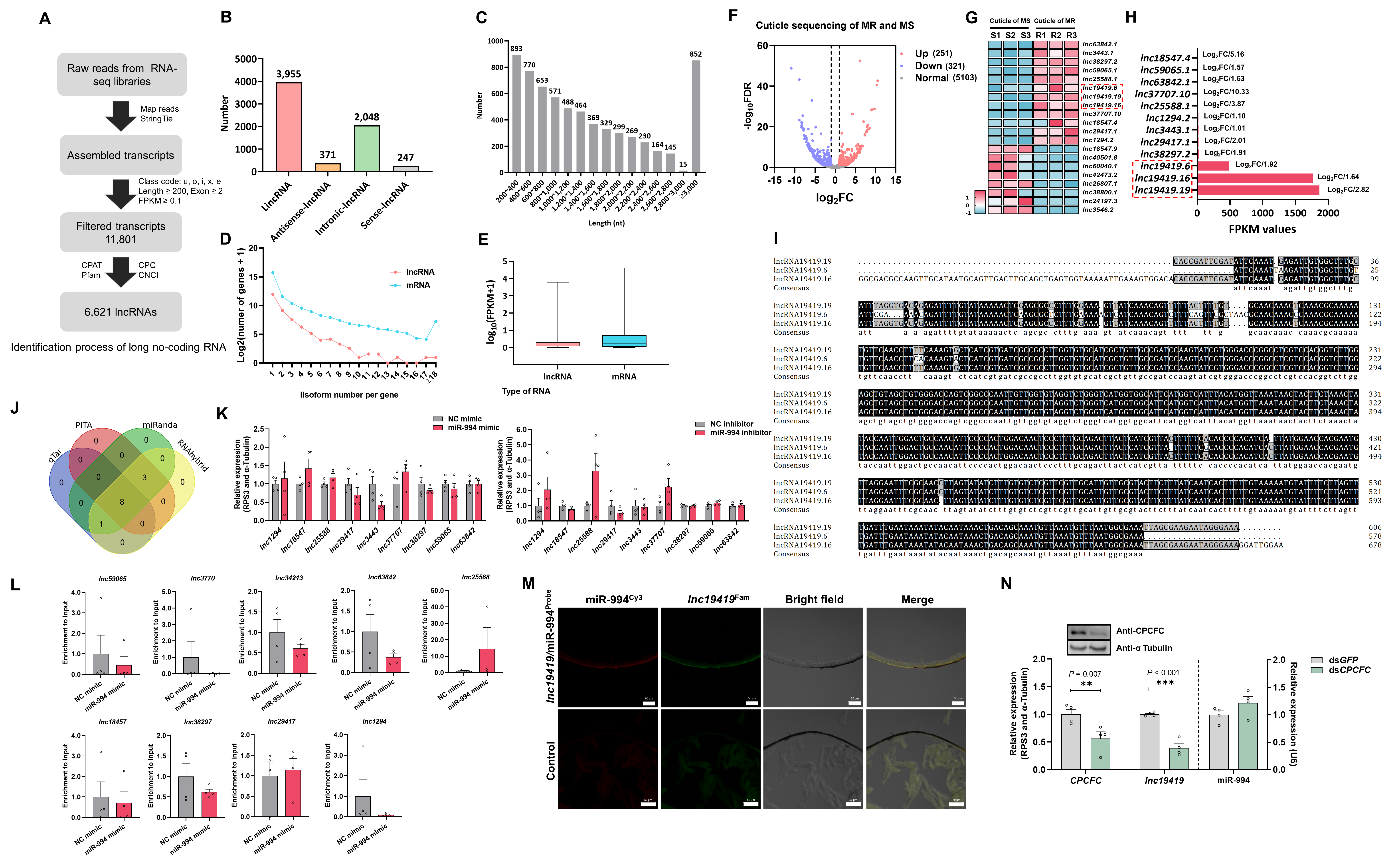


**Fig. S5. The identification of lncRNAs and subsequent bioinformatics analysis.** (A) The identification of lncRNAs from cuticular lncRNA RNA-Seq experiments. (B) Classification of 6,621 identified lncRNAs. (C) The distribution of lncRNA length. (D) Comparative analysis of the number of variable shear isomers of mRNA and lncRNA. (E) Comparative analysis of mRNA and lncRNA expression levels. (F) Differential expression of cuticular lncRNAs between the MR and MS strains. (G) Analysis of differentially expressed lncRNAs, with a threshold FPKM value > 10. Cluster values were analyzed with z-scores (from −1 to 1). (H) FPKM values and fold-change analysis for upregulated lncRNAs. The FPKM values were the means of three biological replicates from the MR strain. (I) Multiple sequence alignment of 3 *lnc19419* transcripts. (J) The potential relationship between the 12 upregulated lncRNA transcripts and miR-994. (K) Expression of lncRNAs following the injection of synthetic miR-994 or an inhibitor. Each sample contained four 5-day-old adult flies. Real-time quantitative PCR data are means ± SEM (*n* = 4). (L) Enrichment analysis (biotin–streptavidin RNA pull-down) of the binding of miR-994 to lncRNAs. Each sample contained eight 5-day-old adult flies. Data are means ± SEM (*n* = 4). (M) Co-localization of miR-994 and *lnc19419* in the pronotum cuticle. The green and red signals (Fam and Cy3 labeling) indicate *lnc19419* and miR-994, respectively. The NC probe was used in the controls. (N) *lnc19419* and miR-994 expression levels analysis after silencing CPCFC. The expression of *lnc19419* and miR-994 was investigated post silencing *CPCFC*. Real-time quantitative PCR data are means ± SEM (*n* = 4). Differences in proportion were compared using Student’s *t*-test (**P* < 0.05, ***P* < 0.01, ****P* < 0.001).
