# Supplementary material for: Cuticular competing endogenous RNAs regulate insecticide penetration and resistance in a major agricultural pest
Source: BMC Biol. 2023 Sep 5;21:187. doi: 10.1186/s12915-023-01694-z (PMC10478477; doi:10.1186/s12915-023-01694-z)
Supplement: Supplementary file 3 — Additional file 3: Tables S1-S3. Table S1. The analysis of detoxification enzyme activities following the injection of synthetic miR-994. Table S2. Toxicity of different insecticides against B. dorsalis strains MS and GX. Table S3. The information of primers, probe and antibody used in this study. [file 12915_2023_1694_MOESM3_ESM.docx]

**Additional File 3: Tables S1-S3. Table S1.** The analysis of detoxification enzyme activities following the injection of synthetic miR-994. **Table S2.** Toxicity of different insecticides against *B. dorsalis* strains MS and GX. **Table S3.** The information of primers, probe and antibody used in this study.

**Table S1.** The analysis of detoxification enzyme activities following the injection of synthetic miR-994.

| **Enzymes** | **Treatment** | **Activity of enzyme** |  | **Treatment** | **Activity of enzyme** |
| --- | --- | --- | --- | --- | --- |
|  | **Post injection 12 h** | |  | **Post injection 24 h** | |
| MFOs | miR-994 mimic | 19.02 ± 11.26 |  | miR-994 mimic | 7.70 ± 0.92 |
|  | NC mimic | 11.70 ± 4.34 |  | NC mimic | 4.51 ± 2.00 |
| CarEs | miR-994 mimic | 34.47 ± 9.81 |  | miR-994 mimic | 36.71 ± 5.85 |
|  | NC mimic | 20.64 ± 2.61 |  | NC mimic | 22.69 ± 4.76 |
| GSTs | miR-994 mimic | 186.27 ± 14.89 |  | miR-994 mimic | 130.75 ± 9.18 |
|  | NC mimic | 161.27 ± 4.52 |  | NC mimic | 117.36 ± 4.69 |

Notes: Units of enzyme activity for multi-function oxidases (MFOs), carboxylesterases (CarEs) and glutathione S-transferases (GSTs) are pmol·min^-1^·mg^-1^, nmol·min^-1^·mg^-1^ and nmol·min^-1^·mg^-1^, respectively.

**Table S2.** Toxicity of different insecticides against *B. dorsalis* strains MS and GX.

| **Pesticide** | **MS** | |  | **GX** | | |
| --- | --- | --- | --- | --- | --- | --- |
|  | **LD_50_ (95% ng/fly)** | **χ^2^** |  | **LD_50_ (95% ng/fly)** | **χ^2^** | **RR** |
| Malathion | 64.37 (56.18–73.40) | 3.63 |  | 170.76 (153.63–189.37) | 2.98 | 2.65 |
| Chlorpyrifos | 4.00 (2.76–5.43) | 6.97 |  | 37.00 (33.12–41.56) | 3.67 | 9.25 |
| Abamectin | 4.85 (4.10–5.78) | 2.61 |  | 8.44 (5.76–12.11) | 8.75 | 1.74 |
| β-cypermethrin | 25.69 (22.24–32.07) | 0.58 |  | 35.88 (30.49–41.74) | 3.44 | 1.39 |

Notes: RR presented the resistant ratio which was insecticides LD_50_ of GX strain/MS strain.

**Table S3.** The information of primers, probe and antibody used in this study.

| **Experiments** | **Primer names** | **Sequence (5′ to 3’)** |
| --- | --- | --- |
| Antibody | CPCFC | MGKPQHLPAAQYPAGVNPQDCPGFPICDNARLHNPQAHWGAPAPAWQPQPQWGAPAPSWQQQQQWGAPAPSWQGAPAPSWQGAPAASAGGDKFPAGVNPHTCPNYPFCDVNAGQHGAVAAPPLPGWTERQYPAGVSAHQCPNFPYCNGSHHHHHH |
|  | α-tubulin | MGSSHHHHHHSSGLVPRGSHMFNTFFSETGAGKHVPRAVFVDLEPTVVDEVRTGTYRQLFHPEQLITGKEDAANNYARGHYTIGKEIVDLVLDRIRKLADQCTGLQGFLIFHSFGGGTGSGFTSLLMERLSVDYGKKSKLEFAIYPAPQVSTAVVEPYNSILTTHTTLEHSDCAFMVDNEAIYDICRRNLDIERPTYTNLNRLIGQIVSSITASLRFDGALNVDLTEFQTNLVPYPRIHFPLVTYAPVISAEKAYHEQLSVAEITNACFEPANQMVKCDPRHGKYMACCMLYRGDVVPKDVNAAIATIKTKRTIQFVDWCPTGFKVGINYQPPTVVPGGDLAKVQRAVCMLSNTTAIAEAWARLDH |
| Heterologous expression | *NdeI*-CPCFC-F | CATATGATGTTCTGCAAATTGGCATT |
|  | *XhoI*-CPCFC-R | CTCGAGCTAGTTGCAGTAGGGGAAGT |
| qPCR analysis | qCPCFC-F | AACAACAGCAACAATGGGGC |
|  | qCPCFC-R | TGACCGGCATTCACATCACA |
|  | qCHS1-F | CTTTGCAATTGGGCGAGGAC |
|  | qCHS1-R | GTGCCCCAAGAGACGACATT |
|  | qCHS1a-F | AATTGCTAAAGATCTTAAAGAGTTGC |
|  | qCHS1a-R | TTGTGTGGATTCGTCGTAGG |
|  | qCHS1b-F | CATCGCAGCCGATCTCAT |
|  | qCHS1b-R | GTTATATTTGTCTTGACGCCCAGT |
|  | qCHS2-F | ATTTTCAGCCTCAAGCCGTA |
|  | qCHS2-R | CGGGACTGCAGAGTACACAA |
|  | qMajor royal jelly protein-F | GGAGAGTGGCGTGGTCTATG |
|  | qMajor royal jelly protein-R | GTTGGCACCAATCGCTCAAG |
|  | qLOC105223504-F | GGTCACTACGCTTGGGTGAA |
|  | qLOC105223504-R | AGTGGCCAATTCGCTTACGA |
|  | qlnc19419-F | TGCAGCTGAGTGGTAAAAAT |
|  | qlnc19419-R | CTGTCACCTAAATGCAAAGCCACAATCTCATTTG |
|  | qlnc63842-F | GTGAACGAGCAAACGAAGCA |
|  | qlnc63842-R | TGTCGTCACTTCCCTTGCAG |
|  | qlnc3443-F | CGACTGGAAGCATCAAGCGA |
|  | qlnc3443-R | GGGGTCACCTAAACCAGACG |
|  | qlnc38297-F | TTTTGCCGACGTTGCATAGC |
|  | qlnc38297-R | ACAAGAAAGCGCGCCAATTT |
|  | qlnc59065-F | AGAGCGGGGAAACGGTATTG |
|  | qlnc59065-R | GGTATACTCGCCGTGTCCAG |
|  | qlnc25588-F | CGCTACTGTGAATGGCGGTA |
|  | qlnc25588-R | ATTCGAAAGCGTGTGCTCCT |
|  | qlnc37707-F | CGAAAAACCGGCAACACCAC |
|  | qlnc37707-R | GCGCACTTCCTGCTTACATT |
|  | qlnc18547-F | TTCTTTAGGAACGCCCCGTC |
|  | qlnc18547-R | ATGTGTTCGCAGTTGTTGCC |
|  | qlnc29417-F | TCGCTGGACACACTCGAAAT |
|  | qlnc29417-R | GCTGCGTGTAATTGGCGTAA |
|  | qlnc1294-F | ACACTAACGCAGCTCTTGCT |
|  | qlnc1294-R | TCCGGCTGCTTGAGTTTGAT |
|  | α-tubulin-F | CGCATTCATGGTTGATAACG |
|  | α-tubulin-R | GGGCACCAAGTTAGTCTGGA |
|  | RPS3-F | TGGATCACCAGAGTGGATCA |
|  | RPS3-R | TAAGTTGACCGGAGGTTTGG |
|  | miR-994 | CGTAAGGAAATAGTAGCCGTGAT |
|  | miR-318 | TCACTGGGCTTTGTTTATCTCA |
|  | miR-6 | CTATCACAGTGGCTGTTCCTTAT |
|  | miR-286 | TGACTAGACCGAACACTCCTGCT |
|  | miR309a-F | TCACTGGGTAAAGTTTGTCCCA |
|  | miR309b-F | GCTCACTGGGTAAAGTTTGTCCT |
|  | miR5a-F | GGAAAGGAACGTTCGTTGTGATAT |
|  | miR5b-F | CCGTATCACAGTGATTTTCCTTGT |
|  | miR11595-F | TATGTTGTTGTCACCGGGAGGACC |
|  | miR4-F | GCATAAAGCTAGACAACCATTGCA |
|  | U6-F | AGGATGACACGCAAAATCGT |
| dsRNA synthesis | dsCHS1-2-F | taatacgactcactatagggATTCTTGATTGCTATGACGG |
|  | dsCHS1-2-R | taatacgactcactatagggATGGTGTTTAATGACTCGGC |
|  | dslnc19419-F | taatacgactcactatagggTCGGCCCAATTGTTGGTGTA |
|  | dslnc19419-R | taatacgactcactatagggCGCAACAATGCAACGAACGA |
|  | dsCPCFC-F | taatacgactcactatagggCGTTGGCCAAACCACAACAT |
|  | dsCPCFC-R | taatacgactcactatagggGGGAATTTATCACCGCCAGC |
|  | dsGFP-F | taatacgactcactatagggCAGTTCTTGTTGAATTAGATG |
|  | dsGFP-R | taatacgactcactatagggTTTGGTTTGTCTCCCATGATG |
| Mutant construction | Mut-CPCFC-F | GCATTTGGAATTTTATTTGAGGAATCTCGAGTCTAGAGTCGACCTGC |
|  | Mut-CPCFC-R | CGTAAACCTTAAAATAAACTCCTTAGAGCTCAGATCTCAGCTGGACG |
|  | Mut-lnc19419-F | GCTTATCAAACAGTTAAAAGAATCTCGAGTCTAGAGTCGACCTGC |
|  | Mut-lnc19419-R | GCAGGTCGACTCTAGACTCGAGATTCTTTTAACTGTTTGATAAGC |
| Probe for *in situ* hybridization. | CPCFC-FAM | AUCGGGAAACCGGGGCAGUCCUGUGGAUUCACGCCAGCUGGGUAC |
|  | lnc19419-FAM | AGUUAUUUAACCAUGUAAAUGACCAUGAAUGCCACCAUGACCCAG |
|  | miR-994-Cy3 | AUCACGGCUACUAUUUCCUUA |
|  | NC | UUGUACUACACAAAAGUACUG |

Notes: T7 promoter sequence: taatacgactcactataggg. FAM and Cy3 are fluorescent labels. The NC probe (no labeling) was from *Caenorhabditis elegans*. The sequence with underline presents the peptide fragment of protein
